# Rab7b regulates dendritic cell migration by linking lysosomes to the actomyosin cytoskeleton

**DOI:** 10.1242/jcs.259221

**Published:** 2021-09-24

**Authors:** Katharina Vestre, Irene Persiconi, Marita Borg Distefano, Nadia Mensali, Noemi Antonella Guadagno, Marine Bretou, Sébastien Wälchli, Catharina Arnold-Schrauf, Oddmund Bakke, Marc Dalod, Ana-Maria Lennon-Dumenil, Cinzia Progida

**Affiliations:** 1 Department of Biosciences, Centre for Immune Regulation, University of Oslo, 0316 Oslo, Norway; 2 Department of Cellular Therapy, the Radium Hospital, Oslo University Hospital, 0379 Oslo, Norway; 3 Institut Curie, Inserm U932, F-75005 Paris, France; 4 VIB-KU Leuven Center for Brain and Disease Research, 3000 Leuven, Belgium; 5 Aix Marseille Univ, CNRS, INSERM, Centre d'Immunologie de Marseille-Luminy, 13288 Marseille, France

**Keywords:** Rab7b, Rab protein, Actomyosin, Cell migration, Dendritic cells

## Abstract

Lysosomal signaling facilitates the migration of immune cells by releasing Ca^2+^ to activate the actin-based motor myosin II at the cell rear. However, how the actomyosin cytoskeleton physically associates to lysosomes is unknown. We have previously identified myosin II as a direct interactor of Rab7b, a small GTPase that mediates the transport from late endosomes/lysosomes to the *trans*-Golgi network (TGN). Here, we show that Rab7b regulates the migration of dendritic cells (DCs) in one- and three-dimensional environments. DCs are immune sentinels that transport antigens from peripheral tissues to lymph nodes to activate T lymphocytes and initiate adaptive immune responses. We found that the lack of Rab7b reduces myosin II light chain phosphorylation and the activation of the transcription factor EB (TFEB), which controls lysosomal signaling and is required for fast DC migration. Furthermore, we demonstrate that Rab7b interacts with the lysosomal Ca^2+^ channel TRPML1 (also known as MCOLN1), enabling the local activation of myosin II at the cell rear. Taken together, our findings identify Rab7b as the missing physical link between lysosomes and the actomyosin cytoskeleton, allowing control of immune cell migration through lysosomal signaling.

This article has an associated First Person interview with the first author of the paper.

## Abstract (precis)

**Summary:** The small GTPase Rab7b bridges the lysosomal Ca^2+^ channel TRPML1 to myosin II, thus enabling the local activation of myosin II at the cell rear and promoting fast migration of dendritic cells.

## INTRODUCTION

Dendritic cells (DCs) are professional antigen-presenting cells that engulf extracellular material in peripheral tissues and transport it to lymph nodes for presentation to T cells. When encountering danger-associated antigens, for example, microbial products, DCs start a maturation program, where they undergo massive phenotypical and functional changes (Banchereau et al., 2000; Reis e Sousa, 2006). In particular, while immature DCs are characterized by a high antigen uptake capacity and a slow intermittent migration mode (Chabaud et al., 2015), mature DCs are less capable of antigen uptake but are highly motile (Vargas et al., 2016). This increase in their migration capacity, together with the upregulation of the CCR7 chemokine receptor at their surface, promotes their migration to lymph nodes for the initiation of adaptive immune responses (Mellman and Steinman, 2001). Therefore, DCs strongly rely on their ability to migrate to exert their immunosurveillance function (Alvarez et al., 2008).

The fast and directional migration that characterizes mature DCs is regulated by lysosomal signaling (Bretou et al., 2017). Lipopolysaccharide (LPS) stimulation in DCs promotes the nuclear translocation of the transcription factor EB (TFEB), the master regulator of lysosome biogenesis and function (Bretou et al., 2017; Sardiello et al., 2009). This results in the expression of a plethora of genes involved in lysosome activity and biogenesis, including the lysosomal Ca^2+^ channel TRPML1 (also known as MCOLN1). Ca^2+^ release through TRPML1 promotes the activity of the actin-based motor myosin II at the cell rear, which regulates fast and directional migration (Bretou et al., 2017). However, it is not known how the actomyosin cytoskeleton physically associates to the lysosomal compartment.

We previously reported that the small GTPase Rab7b, which regulates the transport from late endosomes towards the *trans*-Golgi network (TGN) (Borg Distefano et al., 2018; Progida et al., 2010, 2012), interacts directly with the actin motor protein myosin II (Borg et al., 2014; Distefano et al., 2015). Interestingly, Rab7b was originally identified in DCs (Yang et al., 2004) and later shown to be also expressed upon monocytic and megakaryocytic differentiation (He et al., 2011; Yang et al., 2004). In DCs, Rab7b is strongly upregulated upon LPS-induced maturation, before a gradual downregulation as the cells fully mature (Berg-Larsen et al., 2013), suggesting a possible involvement in some of the initial changes that occur upon maturation.

Here, we investigated the role of Rab7b in DCs. We show that the lack of Rab7b compromises the switch from slow to fast migration that occurs upon DC maturation, with antigen uptake remaining unaffected. We further highlight that lysosomal signaling and myosin II activity are reduced in Rab7b-knockout (KO) DCs. Finally, we demonstrate that Rab7b interacts with the lysosomal Ca^2+^ channel TRPML1, bridging it to myosin II for the local activation at the cell rear. These results strongly suggest that Rab7b acts as the missing physical link between lysosomes and the actomyosin cytoskeleton, thereby promoting fast DC migration.

## RESULTS

### Rab7b is needed for actomyosin polarization in mature DCs

**Fig. 1.**
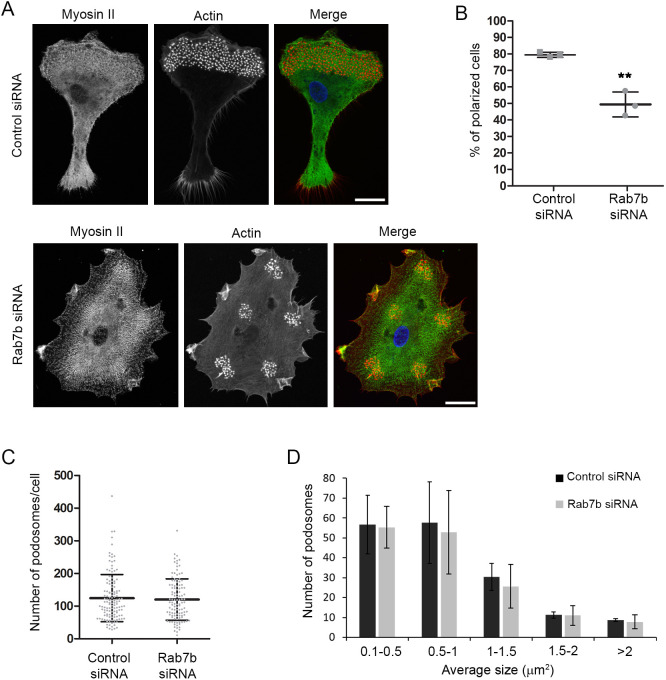
**Rab7b is required for DC polarization.** (A) MDDCs were transfected by electroporation with either control siRNA or Rab7b siRNA, and stimulated with LPS for 48 h. Thereafter, DCs were plated on PLL-coated coverslips and left to adhere for additional 24 h, before fixation and immunostaining with anti-myosin II (green). Actin was labeled with Rhodamine-conjugated phalloidin (red) and nuclei with Hoechst 33258 (blue). Images represent maximum intensity projections of Z stacks. Scale bars: 10 µm. (B) Quantification of the percentage of polarized cells. Polarized cells have a clear podosome-rich leading edge and a trailing edge devoid of podosomes. Cells with podosomes equally distributed and with no distinction between leading and trailing edges are accounted as not polarized. Data represent the mean±s.d. of three independent experiments (*n*>145). **P<0.005 (two-tailed unpaired Student's *t*-test). (C,D) Quantification of the number (C) and size (D) of podosomes per cell. The graphs show the mean±s.d. from three independent experiments (*n*>120).

Rab7b is highly expressed in DCs, and strongly upregulated upon LPS-induced maturation (Berg-Larsen et al., 2013; Progida et al., 2010). However, it is currently unknown what the role of this small GTPase in maturing DCs is. To assess the function of Rab7b in DCs, we first analyzed the intracellular localization of the endogenous protein in monocyte-derived human DCs (MDDCs). As shown in Fig. 1A and Fig. S1A, Rab7b localizes to lysosomes but not to early endosomes nor the TGN in MDDCs. We also found Rab7b to colocalize with its interactor myosin II. We therefore next investigated how the actomyosin organization was affected in cells depleted of Rab7b. MDDCs were transfected with siRNA targeting Rab7b, stimulated with LPS, seeded on poly-L-lysine (PLL)-coated coverslips, were fixed and stained with the actin marker phalloidin and an antibody against myosin II. Intriguingly, while control cells were clearly organized in a polarized and directional manner, with actin-rich structures called podosomes only at the leading edge, DCs depleted of Rab7b lacked polarization, and podosome orientation was altered (Fig. 1A,B). Interestingly, the altered orientation and distribution of podosomes observed upon Rab7b knockdown resembled the ones observed in immature DCs (Fig. S1B). As we did not detect any significant differences in the size or number of podosomes per cell in cells depleted of Rab7b compared to control cells (Fig. 1C,D), we conclude that Rab7b does not affect podosome formation, but rather their distribution and the polarization of mature human DCs.

### Depletion of Rab7b does not prevent DC maturation or antigen presentation ability

**Fig. 2.**
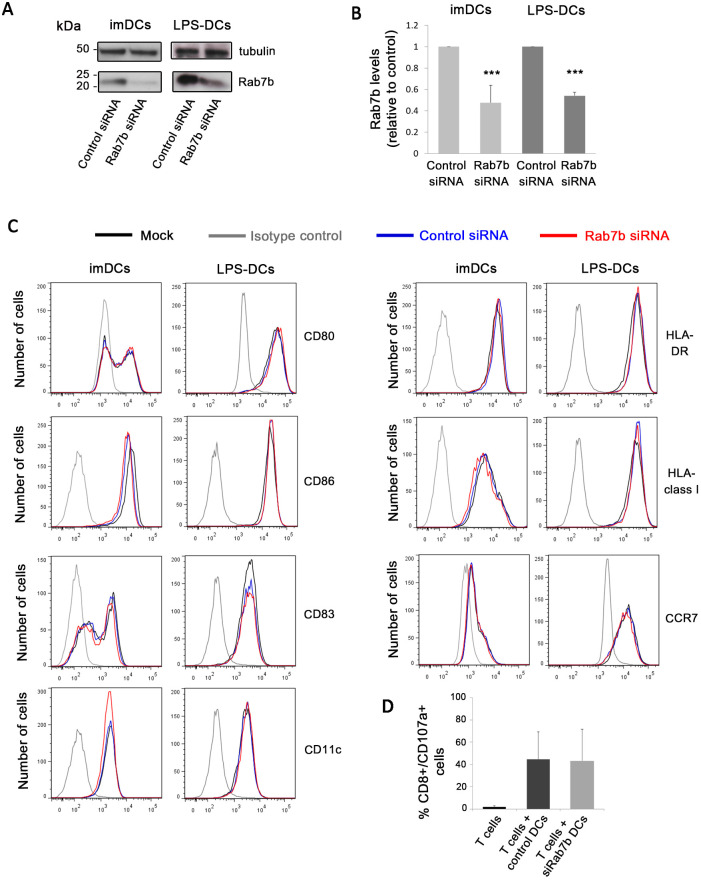
**Rab7b depletion does not affect DC maturation and antigen presentation ability.** (A) MDDCs were transfected by electroporation with either control siRNA or Rab7b siRNA. 18 h (for immature DCs; imDCs) or 48 h (for LPS-DCs) after transfection the DCs were harvested, lysed and subjected to western blot analysis with antibodies against Rab7b and tubulin as a loading control. (B) Quantification of Rab7b levels in MDDCs silenced with control siRNA or Rab7b siRNA. The intensity of the bands from western blots was quantified using ImageQuant, and the level of Rab7b was normalized to the amount of tubulin. Data represent the mean±s.d. of three independent experiments. ***P<0.0001 (two-tailed unpaired Student's *t*-test). (C) FACS analysis of the surface expression markers CD80, CD86 CD83 and CD11c (left panels), and HLA-DR, HLA class I and CCR7 (right panels). A representative histogram overlay is shown for each marker. The black line represents mock electroporated (without siRNA) MDDCs, the blue line the control siRNA and the red line the Rab7b siRNA electroporated MDDCs. The gray line corresponds to isotype antibodies. The *x-*axis represents the mean fluorescence intensity of the conjugated markers indicated for each histogram. The histograms are representative examples from one out of three independent experiments. All experiments were repeated three independent times. (D) Radium-1 TCR-expressing T cells were stimulated for 5 h with either control siRNA- or Rab7b siRNA-treated DCs loaded with a specific 19-mer peptide encoded by the *TGFBR2* frameshift mutation. An anti-CD107a antibody was used to assess the amount of degranulation by CD8+ cytotoxic T cells specifically activated by DCs. Data represents the mean±s.d. of three independent experiments.

To investigate whether the altered DC polarization upon Rab7b depletion was a consequence of defective maturation rather than a specific effect on the actomyosin cytoskeleton, we measured the expression of maturation markers on the DC surface by flow cytometry (Banchereau et al., 2000). Both immature and mature LPS-treated DCs were efficiently depleted of Rab7b (Fig. 2A,B). We found no significant difference in surface expression of maturational markers between Rab7b siRNA-treated and control siRNA-treated DCs (Fig. 2C). Indeed, the levels of the co-stimulatory molecules CD80 and CD86, as well as human leukocyte antigen (HLA)-class I and HLA-DR, remained unchanged upon Rab7b knockdown. Likewise, the surface levels of the immunoregulatory molecule CD83, the chemokine receptor CCR7 and the DC marker CD11c (also known as ITGAX) were unaffected (Fig. 2C). We excluded that the electroporation or the depletion of Rab7b by itself initiated the DC maturation process, as immature DCs depleted of Rab7b, similar to immature control cells, displayed lower levels of the surface markers of mature DCs, like CD80, CD86 and CD83 (Fig. 2C). Thus, Rab7b does not affect the maturation of DCs as Rab7b-depleted DCs show a normal maturation pattern upon LPS treatment.

Phenotypic markers are commonly used to assess DCs maturation (Banchereau et al., 2000; Manh et al., 2013). However, this assessment is not sufficient to determine whether DCs have acquired the ability to present antigens. Therefore, we also investigated whether depletion of Rab7b affects the ability of DCs to present antigens to T cells *in vitro* by performing a CD107a (also known as LAMP1) mobilization assay. DCs isolated from healthy donors were successfully depleted for Rab7b (Fig. S2A,B), loaded with a specific cancer peptide (a TGFBR2 frameshift mutation-derived epitope), and further incubated with autologous T cells transfected with the validated cognate T cell receptor (TCR), Radium-1 (Inderberg et al., 2017). The amount of specifically stimulated CD8+ T cells, monitored by measuring the levels of the degranulation marker CD107a, was measured by flow cytometry. As shown in Fig. 2D, depletion of Rab7b did not affect the antigen presentation abilities of mature DCs, as both the control siRNA- and the Rab7b siRNA-treated DCs stimulated T cell activation to the same extent. Taken together, our results indicate that although Rab7b regulates actin polarization in DCs, it does not play any role in their maturation and ability to present antigens to T lymphocytes.

### Rab7b depletion prevents fast and persistent DC migration

**Fig. 3.**
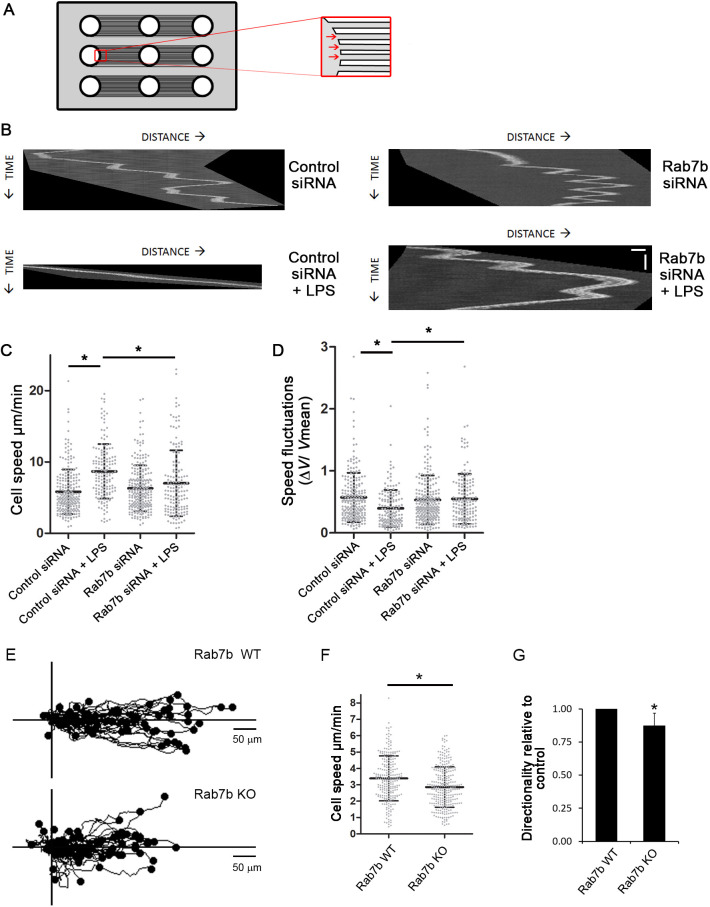
**Rab7b is required for fast and persistent migration of LPS-DCs.** (A) Scheme of a micro-fabricated device used to study DC motility under confinement. Cells are loaded in the loading chambers, and spontaneously enter into the microchannels (inset with arrows indicating entry points). (B) BMDCs were either mock treated or LPS-treated for 20 min, before transfection with either control siRNA or Rab7b siRNA. DCs were loaded in 5×5 µm micro-fabricated channels and imaged for 20 h in an epifluorescence Nikon TiE microscope equipped with a cooled CCD camera, using a 10× objective and acquiring one transmission phase image every 2 min. Representative kymographs are shown for DCs treated with either control siRNA, with or without LPS (left panels), or Rab7b siRNA, with or without LPS (right panels). Scale bars: horizontal (distance), 20 µm; vertical (time), 30 min. (C) Quantification of the mean±s.d cell speed (µm/min). *n*>150, three independent experiments. *P<0.05 (two-tailed unpaired Student's *t*-test). (D) Quantification of mean±s.d. speed fluctuations [calculated as s.d./mean instantaneous speed (Chabaud et al., 2015; Faure-Andre et al., 2008)]. *n*>150, three independent experiments. *P<0.05 (two-tailed unpaired Student's *t*-test). (E) Chemotactic response of LPS-DCs embedded in a collagen gel containing a CCL21 gradient. The plot represents movement in the *x-* and *y*-direction of single cells, each track starting at distance 0, from one representative experiment. (F) Quantification of the mean cell speed of WT and Rab7b KO LPS-DCs. Data represents the mean±s.d. of four independent experiments (*n*=238 and 284 cells for WT and Rab7b-KO, respectively). *P<0.05 (two-tailed paired Student's *t-*test). (G) Quantification of the cell persistency of WT and Rab7b KO LPS-DCs. Cell persistency was calculated by dividing the Euclidian distance with the accumulated distance of each cell trajectory, and is presented relative to WT. Data represents the mean±s.d. of four independent experiments (*n*=238 and 284 cells for WT and Rab7b-KO, respectively). *P<0.05 (two-tailed unpaired Student's *t*-test).

As cell polarization is important for cell migration, we next evaluated whether Rab7b knockdown also affects DC motility. DC migration is strongly dependent on external geometry, and DCs migrate faster in confined environments (Heuze et al., 2013; Lammermann et al., 2008). To study the effect of Rab7b depletion on DC migration under confinement, we used micro-fabricated channels (Fig. 3A). These experiments were performed with bone marrow-derived murine DCs (BMDCs), as previously described (Bretou et al., 2017; Chabaud et al., 2015; Vargas et al., 2016, 2014). Similar to human MDDCs, BMDCs were successfully depleted for Rab7b, and the depletion did not affect their maturation (Fig. S2C,D). BMDCs transfected with control siRNA or siRNA targeting Rab7b were loaded into microchannels and imaged overnight. Our results showed that for cells transfected with the control siRNA, immature DCs were slower (mean speed 4.8 µm/min) than the mature LPS-stimulated DCs (LPS-DCs; mean speed 8.4 µm/min) (Fig. 3B,C), as expected. However, cells depleted of Rab7b failed to speed up after addition of LPS (Fig. 3B,C).

While immature DCs are known to change direction frequently, undergoing important speed fluctuations during motion, mature DCs are more persistent (Chabaud et al., 2015; Vargas et al., 2016). Interestingly, upon depletion of Rab7b, LPS-DCs still behave like immature DCs, as they have low speed and significantly higher local speed variations compared to control cells, indicating that DCs depleted of Rab7b change direction more frequently while migrating in microchannels (Fig. 3D). These results point to a role for Rab7b in DC polarization and migration, suggesting that the lack of this small GTPase prevents the switch to the faster and more persistent locomotion typical of mature DCs.

To further explore the role of Rab7b in DC migration, we generated a CD11c conditional knockout (KO) mouse model for this small GTPase. We then investigated whether the Rab7b-dependent migration defects observed in LPS-DCs affected their chemotactic migration. When exposed to the chemokine CCL21 *in vivo*, DCs increase their persistency and are guided towards lymphatic vessels (Weber et al., 2013). To model this process, mature LPS-DCs from conditional KO mice were embedded in collagen gels and left to migrate in the presence of a CCL21 gradient. Consistent with our previous results, we found that Rab7b KO DC migration speed was significantly decreased. Indeed, Rab7b KO DCs were over 20% slower than wild-type (WT) cells (Fig. 3E,F; Movie 1). Similarly, Rab7b KO LPS-DCs were also significantly less persistent in comparison to the WT cells (Fig. 3G). Taken together, these results demonstrate that Rab7b is needed for mature DCs to switch to a fast and directional migration mode.

### Rab7b affects actomyosin distribution and macropinocytosis in LPS-DCs

**Fig. 4.**
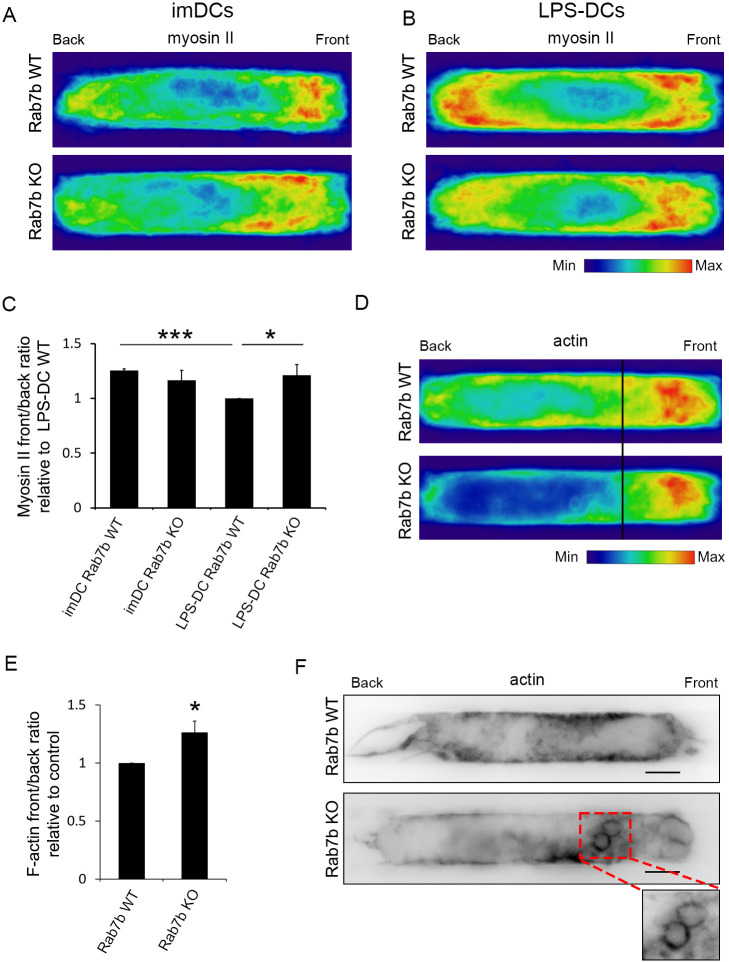
**Rab7b affects actomyosin distribution.** (A,B) LPS-DCs were loaded in 5×8 µm micro-fabricated channels, fixed after 16 h and stained with an antibody against myosin II. The intensity of each cell for each condition was averaged into a single density map. One representative experiment out of three is shown. imDCs, immature DCs. (C) Quantification of the myosin front-to-back ratio. Data represents the mean±s.d. of three independent experiments (*n*>48 cells for each condition). *P<0.05; ***P<0.001 (two-tailed unpaired Student's *t*-test). (D) LPS-DCs were loaded in 5×8 µm micro-fabricated channels, fixed after 16 h and labeled with Rhodamine-conjugated phalloidin to visualize actin. The intensity of each cell for each condition was averaged into a single density map. One representative experiment out of three is shown. (E) Quantification of the F-actin front-to-back rratio relative to WT. Data represents the mean±s.d. of three independent experiments (*n*=51 and 57 cells for WT and Rab7b KO, respectively). *P<0.05 (two-tailed unpaired Student's *t*-test). (F) LPS-DCs were loaded in 5×8 µm micro-fabricated channels, fixed after 16 h and labeled with Rhodamine-conjugated phalloidin to visualize actin. Representative images from one out of three independent experiments are shown. Images are inverted to improve visualization. Scale bars: 5 µm.

The migration of DCs in confined environments depends on contractile forces driven by myosin II (Chabaud et al., 2015; Lammermann et al., 2008). To promote fast migration, mature DCs increase the amount of actin and myosin at the cell rear, while immature DCs have more actin and myosin at the cell front to perform macropinocytosis (Chabaud et al., 2015; Vargas et al., 2016). Since our data indicate that Rab7b is important for polarization of DCs, we speculated that the decreased migratory ability of DCs lacking Rab7b might be a consequence of the altered actomyosin distribution. We therefore compared myosin II distribution in immature and mature WT and Rab7b KO DCs migrating in micro-fabricated channels. The analysis of density maps of the mean myosin II distribution showed that Rab7b KO cells, in contrast to WT cells, failed to increase the amount of myosin II at the cell rear after addition of LPS (Fig. 4A–C). While the front-to-back ratio for myosin II was not significantly different between WT and KO immature cells, the front-to-back ratio in Rab7b KO LPS-DCs was 32% higher than in the WT LPS-DCs (Fig. 4C).

Having observed that the myosin II distribution is altered in LPS-treated Rab7b KO DCs, we next looked at whether the actin distribution was affected in a similar way in these cells. In line with our results for myosin II, density maps of the mean actin distribution showed that the fraction of actin located at the cell front in LPS-DCs was almost 30% higher in the Rab7b KO cells than in the WT cells (Fig. 4D,E). Intriguingly, this distribution is similar to the distribution in immature DCs during phases of slow locomotion (Chabaud et al., 2015; Vargas et al., 2016).

**Fig. 5.**
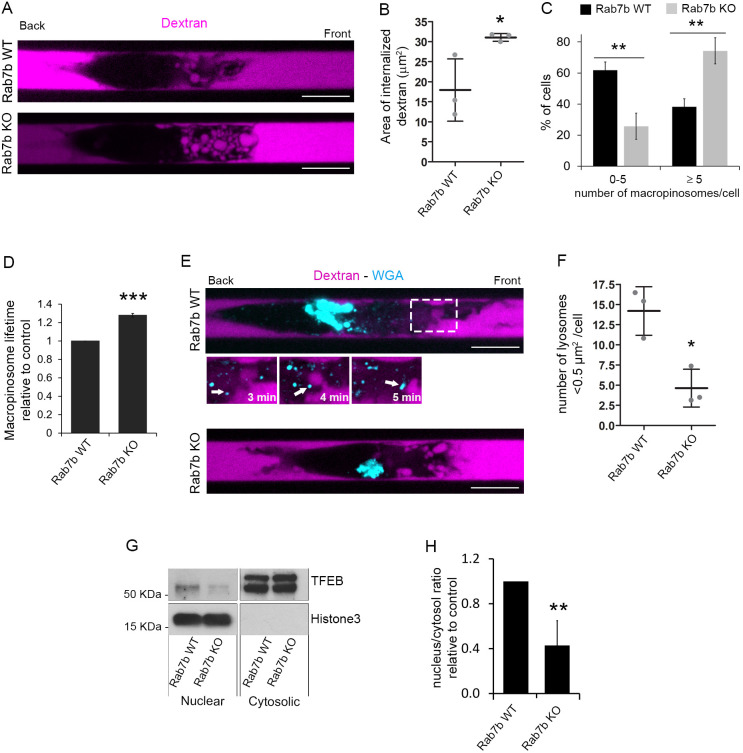
**Rab7b affects macropinocytosis and lysosome signaling.** (A) LPS-DCs were loaded in 5×8 µm micro-fabricated channels. After 16 h, the channels were filled with 10 kDa Alexa Fluor 647-conjugated dextran (magenta) and the cells were imaged 30 min later. Representative images of live WT and Rab7b KO cells are shown. Scale bars: 10 µm. (B) Quantification of the area of internalized dextran in WT and Rab7b KO cells. The graph represents the mean±s.d. of three independent experiments (*n*=39 and 32 cells for WT and Rab7b-KO, respectively). *P<0.05 (two-tailed unpaired Student's *t*-test). (C) Distribution of macropinosome numbers for WT and Rab7b KO cells from three independent experiments. Data represents the mean±s.d. (*n*=39 and 32 cells for WT and Rab7b KO, respectively). **P<0.01 (two-tailed unpaired Student's *t*-test). (D) Quantification of the lifetime of macropinosomes in WT and Rab7b KO cells. Data represents the mean±s.d. of three independent experiments (>100 tracked macropinosomes per condition, *n*=23 and 28 cells for WT and Rab7b KO, respectively). ***P<0.001 (two-tailed unpaired Student's *t-*test). (E) Spinning disk images of live WT and Rab7b KO LPS-DCs stained with Alexa Fluor 594-conjugated WGA to label lysosomes (cyan) and loaded in 5×8 µm micro-fabricated channels filled with 10 kDa Alexa Fluor 647-conjugated dextran (magenta). Representative images from one out of three independent experiments are shown. Scale bar: 10 µm. The magnified area shows a lysosome (arrow) moving towards a macropinosome in a control cell. Image contrast has been increased to improve visualization. (F) Quantification of the number of lysosomes with area <0.5 µm^2^ per cell. Data represents the mean±s.d. of three independent experiments (*n*=29 cells for WT and Rab7b-KO). *P<0.05 (two-tailed unpaired Student's *t*-test). (G) BMDCs from WT and Rab7b KO mice were pulsed with 100 ng/ml LPS for 30 min and lysed after 6 h. Cytosolic and nuclear fractions were subjected to immunoblotting analysis with the indicated antibodies. Histone 3 was used as control of the cytosolic and nuclear fraction separation. (H) The graph shows the quantification of the nucleus-to-cytosol ratio for TFEB in Rab7b-KO DCs relative to WT. Data represents the mean±s.d. of four independent experiments. **P<0.01 (two-tailed unpaired Student's *t*-test).

Immature DCs are characterized by a high antigen uptake capacity, and they sample their environment by engulfing large amounts of extracellular material using macropinocytosis. The enrichment of actin and myosin at the cell front is typical for DCs performing macropinocytosis, and, in line with this, the actin at the cell front in Rab7b KO DCs was mainly localized in ruffles and around macropinosomes (Fig. 4F). We therefore verified whether the increased actin concentration at the front of Rab7b-KO DCs was the result of increased macropinocytic activity. For this, LPS-DCs were imaged in microchannels filled with fluorescently labeled dextran to visualize macropinosomes. Similar to what was found in previous studies (Chabaud et al., 2015), we observed large dextran-containing macropinosomes that formed at the front of the DCs (Fig. 5A; Movie 2). As expected for LPS-DCs, the majority of WT cells displayed few or no macropinosomes. However, in the Rab7b-KO LPS-DCs, both the number of macropinosomes and the area of internalized dextran were significantly increased (Fig. 5A–C). Furthermore, while the macropinosomes in the WT cells disappeared quickly after they were formed, the macropinosomes in the Rab7b KO DCs persisted for a longer time. Indeed, the average lifetime of single macropinosomes in Rab7b KO LPS-DCs was 28% longer than in WT LPS-DCs (Fig. 5D; Movie 2). Altogether, these results indicate that the decreased motility of Rab7b KO LPS-DCs is associated with their sustained macropinocytic activity.

### Lysosome signaling is inhibited in Rab7b KO DCs

Antigens taken up by macropinocytosis are delivered to lysosomes, which become more active during maturation (Trombetta et al., 2003). Previous studies have shown that the inhibition of macropinocytosis after DC maturation activates lysosomal signaling, which in turn is important for controlling the changes in DC migration by facilitating the formation and/or maintenance of actomyosin at the cell rear (Bretou et al., 2017). Since Rab7b is involved in lysosomal transport (Progida et al., 2010) and also affects DC migration and macropinocytic activity, we next analyzed lysosome distribution in Rab7b KO DCs. LPS-DCs were incubated with fluorescent wheat germ agglutinin (WGA) to label lysosomes, as previously described (Bretou et al., 2017), before loading the cells into dextran-filled microchannels. Consistent with what has been seen in previous studies (Bretou et al., 2017), we observed a large cluster of lysosomes located at the rear in WT LPS-DCs. A similar distribution was also observed in Rab7b KO cells (Fig. 5E), and no differences in either size or distribution of lysosomes or orientation of the microtubule-organizing center (MTOC) was detected between WT and Rab7b KO DCs (Fig. S4A–E). However, while WT cells also had several small and highly dynamic lysosomes that mainly moved between the main cluster and the cell front making contact with the macropinosomes, Rab7b KO cells were devoid of this lysosomal population (Fig. 5E; Movie 3). Indeed, the number of small lysosomes (with an area <0.5 µm^2^) per cell in WT LPS-DCs was over three times higher than in Rab7b KO cells (Fig. 5E,F).

This result suggests that Rab7b influences lysosome dynamics in DCs. We thus investigated whether it also affects the nuclear translocation of the transcription factor TFEB, the master regulator of lysosome biogenesis and function (Bretou et al., 2017; Sardiello et al., 2009). LPS stimulation in DCs promotes the nuclear translocation of TFEB, which is required for triggering the fast and directional migration that characterizes mature DCs (Bretou et al., 2017). Interestingly, the lack of Rab7b reduces by ∼60% the fraction of TFEB in the nucleus in LPS-DCs (Fig. 5G,H). Altogether, our results indicate that Rab7b modulates lysosomal signaling through TFEB, which is in turn required for the re-organization of the actin cytoskeleton and fast migration of mature DCs.

### Lack of Rab7b reduces myosin II phosphorylation

**Fig. 6.**
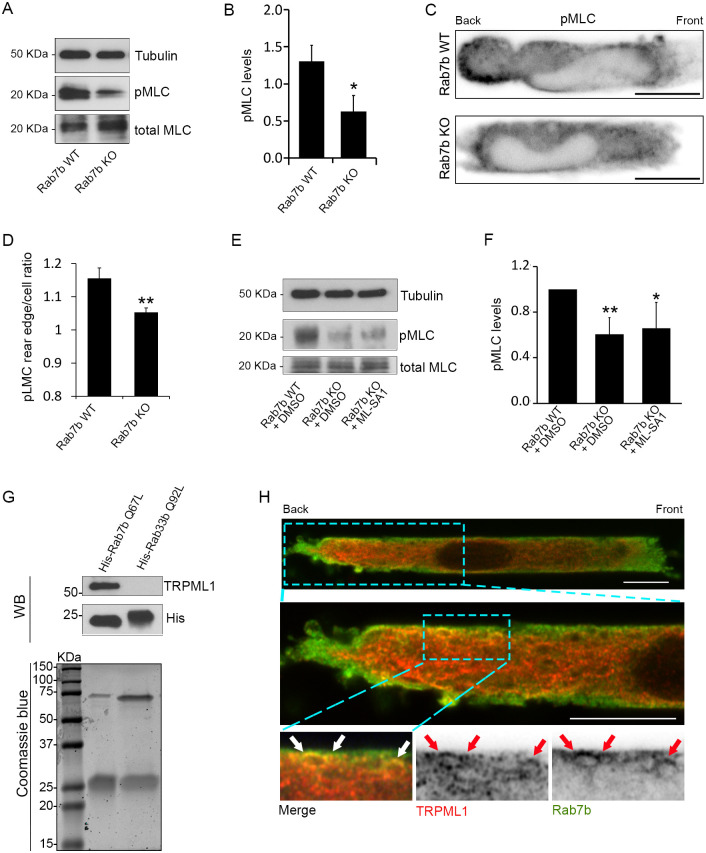
**Rab7b regulates myosin II activation and interacts with the lysosomal Ca^2+^ channel TRPML1.** (A) Lysates from BMDCs from WT and Rab7b KO mice pulsed with 100 ng/ml LPS for 30 min were subjected to immunoblotting analysis with the indicated antibodies. Tubulin was used as loading control. (B) The graph shows the quantification of phosphorylated myosin light chain (pMLC) levels in WT and Rab7b KO DCs normalized to the tubulin levels. Data represents the mean±s.d. of four independent experiments. *P<0.05 (two-tailed unpaired Student's *t*-test). (C) LPS-DCs were loaded in 5×8 µm micro-fabricated channels, fixed after 16 h and stained with and antibody against phosphorylated myosin light chain (pMLC). Representative images from one out of three independent experiments are shown. Images are inverted to improve visualization. Scale bars: 10 µm. (D) Quantification of the pMLC rear edge-to-cell ratio. Data represents the mean±s.d. of three independent experiments (*n*=72 and 84 cells for WT and Rab7b-KO, respectively). **P<0.01 (two-tailed unpaired Student's *t*-test). (E) BMDCs from WT and Rab7b KO mice pulsed with 100 ng/ml LPS for 30 min were treated with either DMSO or ML-SA1 20 µM overnight and then lysed. Lysates were subjected to immunoblotting analysis with the indicated antibodies. Tubulin was used as loading control. (F) The graph shows the quantification of pMLC levels in WT and Rab7b KO DCs normalized to the tubulin levels. Data represents the mean±s.d. of five independent experiments. *P<0.05; **P<0.01 (two-tailed unpaired Student's *t*-test). (G) Lower panel, Coomassie Blue staining of bacterially expressed His–Rab7b Q67L (constitutively active mutant) and His–Rab33b Q92L (constitutively active mutant) purified using Ni-NTA agarose matrix. Upper panel: bacterially expressed and purified His–Rab7b Q67L and His–Rab33b Q92L were incubated with lysates from LPS-treated MDDCs. Proteins were pulled down using cobalt-coated magnetic beads and subjected to western blot (WB) analysis using antibodies against His and TRPML1. (H) LPS-MDDCs were loaded in 5×8 µm micro-fabricated channels, fixed after 16 h and stained with antibodies against TRPML1 (red) and Rab7b (green). The red and white arrows indicate colocalization between TRPML1 and Rab7b. Scale bars: 10 µm. The magnified images were acquired using super-resolution mode.

The lysosomal signaling pathway activated by TFEB also leads to increased phosphorylation of myosin II, a direct interaction partner of Rab7b. Indeed, TFEB triggers the activation of myosin light-chain kinase, which is responsible for phosphorylation of the light chain of myosin II (MLC; herein referring to MYL9) (Bretou et al., 2017). The phosphorylation of MLC is important for the regulation of actin cytoskeletal dynamics, therefore, we investigated whether Rab7b influences MLC phosphorylation in DCs. Our data confirmed that the proportion of phosphorylated MLC is reduced by ∼50% in Rab7b KO cells compared to WT cells (Fig. 6A,B). Immunofluorescence analysis further revealed that phosphorylated MLC (pMLC) is enriched at the cell rear edge of WT but not Rab7b KO DCs migrating in microchannels (Fig. 6C,D). This suggests that Rab7b is involved in the activation of myosin II at the cell rear that is responsible for triggering fast motility.

The phosphorylation of MLC triggered by TFEB is dependent on the local release of Ca^2+^ from the lysosomes via the transient receptor potential cation channel, mucolipin subfamily, member 1 (TRPML1) (Bretou et al., 2017). To investigate whether the reduced MLC phosphorylation in Rab7b KO LPS-DCs was caused by the inactivation of TRPML1 or by the impaired Rab7b-mediated recruitment of myosin II to the lysosomes, we treated the cells with ML-SA1, an agonist of the TRPML1 channel (Shen et al., 2012). As shown in Fig. 6E,F, stimulation of lysosomal Ca^2+^ release by ML-SA1 in Rab7b KO DCs was not sufficient to restore MLC phosphorylation. As Rab7b interacts directly with myosin II (Borg et al., 2014; Distefano et al., 2015), this result supports a model where, in the absence of Rab7b, the localized Ca^2+^ release from the lysosomes cannot activate myosin II as the recruitment of this motor protein to the lysosomes is prevented. As a result, actin localization at the rear of LPS-DCs is hampered, thereby preventing fast and persistent DC migration. Intriguingly, and in line with this model, we demonstrated that His-tagged Rab7b pulled down TRPML1 from total cell extracts (Fig. 6G), and the two proteins colocalize at the cell rear in migrating DCs (Fig. 6H).

Altogether, these data point to a role of Rab7b in the regulation of DC migration by physically linking actomyosin to lysosomes.

## DISCUSSION

After antigen uptake, DCs modify their migratory behavior, triggering a fast and directed migration mode. Lysosomal signaling is involved in this process, by stimulating local Ca^2+^ release and the activity of myosin II. How the actomyosin cytoskeleton physically associates to lysosomes has nonetheless remained elusive. Here, we show that Rab7b is the missing link between lysosomes and the actomyosin cytoskeleton, controlling the fast migration of DCs through lysosomal signaling.

Rab7b is a small GTPase that regulates the transport from late endosomes/lysosomes to the TGN (Progida et al., 2010, 2012). It is highly expressed in DCs, with a burst of expression upon LPS-induced maturation (Berg-Larsen et al., 2013; Yang et al., 2004). Why maturing DCs upregulate the expression of this small GTPase is, however, unknown. Here, we found that DCs depleted of Rab7b are significantly less polarized (Fig. 1), demonstrating that this small GTPase is important for the polarization of the mature DCs. In line with this, Rab7b KO LPS-DCs migrating in microchannels have more actin and myosin in the front compared to WT cells (Fig. 4), indicating that, in the absence of Rab7b, mature DCs fail to re-orient their actin and myosin properly.

This actomyosin reorganization is essential for the ability of mature DCs to migrate fast and efficiently towards lymph nodes. The pool of actin and myosin at the cell front is indeed associated with slow motility, which is mainly observed in immature DCs and is responsible for membrane ruffling and macropinosome formation. On the contrary, the presence of an actomyosin pool at the cell rear characterizes mature DCs and their ability to migrate faster and more persistently (Vargas et al., 2016). In agreement with this, the retained actin distribution at the front of DCs lacking Rab7b is consistent with their increased macropinocytic activity compared to WT cells (Fig. 5A–D), as well as with their slow and less persistent motility (Fig. 3). As depletion of Rab7b causes a significant decrease in the speed and directionality of DCs, our findings demonstrate that this small GTPase, by regulating actomyosin distribution, is important for the reduction of macropinocytic activity and the acquisition of the fast migratory ability of mature DCs. This is supported by the defective polarization of DCs when Rab7b is silenced; if the cells cannot polarize properly, migration and directionality is also impaired (Danuser et al., 2013).

Lysosomal signaling plays a crucial role in triggering the re-organization of the actomyosin cytoskeleton at the cell rear that is necessary for the fast chemotactic DC migration upon LPS sensing. These signaling events involve translocation of the transcription factor TFEB, a master regulator of lysosome biogenesis and function (Sardiello et al., 2009), to the nucleus (Bretou et al., 2017). As Rab7b is required for proper lysosome function (Progida et al., 2010), it is not surprising that it is involved in the nuclear translocation of TFEB. Indeed, the lack of Rab7b alters lysosome dynamics and reduces TFEB translocation to the nucleus in the LPS-DCs (Fig. 5E–H). This suggests that the failure in Rab7b KO cells in triggering fast migration is a result of impaired lysosomal signaling through TFEB.

**Fig. 7.**
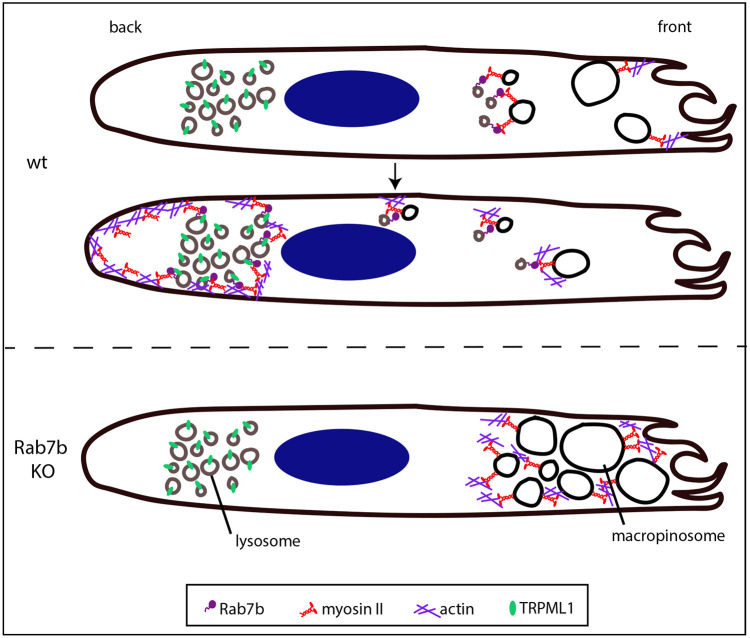
**Model to illustrate the role of Rab7b in DCs.** Upon microbial sensing, DCs increase their migration ability and decrease their capacity of antigen uptake by macropinocytosis. Upregulation of Rab7b promotes this switch by recruiting myosin II from macropinosomes to late endocytic compartments, bringing the motor in close proximity to TRPML1, which activates myosin II at the cell rear and promotes fast DC motility. In the absence of Rab7b, myosin II is retained on macropinosomes at the front of the cells, promoting formation of large macropinosomes and slowing down migration.

How does Rab7b regulate TFEB translocation? DCs take up foreign material using macropinocytosis, and the ingested antigens are delivered to lysosomes (Norbury, 2006). Previous studies have shown that myosin II is important for the trafficking of macropinosomes towards the cell rear, and that it promotes the delivery of antigens to endolysosomal compartments (Chabaud et al., 2015). Since Rab7b interacts directly with myosin II (Borg et al., 2014) and has a role in the trafficking of endolysosomal compartments (Progida et al., 2010), it is likely that Rab7b, by recruiting myosin II from macropinosomes to late endocytic compartments, mediates the transport of internalized material to lysosomes. According to this model, in absence of Rab7b, myosin II is retained on macropinosomes at the front of the cells, promoting formation of large macropinosomes and slowing down migration (Fig. 7). The sustained macropinocytosis activity inhibits TFEB nuclear translocation as this transcription factor is activated by the downregulation of macropinocytosis (Bretou et al., 2017). In line with this, stimulation of lysosomal Ca^2+^ release, which is known to promote myosin phosphorylation, thereby triggering localized actomyosin contractility at the rear of mature DCs, is not able to rescue MLC phosphorylation defects in Rab7b KO DCs (Fig. 6A–F). Since we reveal that Rab7b interacts with the lysosomal Ca^2+^ channel TRPML1, this indicates that Rab7b brings its effector myosin II in close proximity to TRPML1 to be activated by the localized Ca^2+^ release from this channel.

In conclusion, we have identified Rab7b as a physical link between lysosomes and the actomyosin cytoskeleton in DCs, and found that this small GTPase is critical for proper polarization and the fast migratory ability of these immune cells by coordinating lysosomal signaling and actomyosin cytoskeleton reorganization.

## MATERIALS AND METHODS

### Mice and cells

C57BL/6 mice bearing a *Rab7b*-KO (*Rab7b^tm2Ciphe^*) allele specifically in CD11c^+^ cells, including in BMDCs, corresponding to the deletion of exon 5 of the *Rab7b* gene, have been generated by crossing *Rab7b*-floxed/reporter (*Rab7b^tm1Ciphe^*) C57BL/6 mice with CD11c-Cre mice [B6.Cg-Tg(Itgax-cre)1-1Reiz/J] (Fig. S3). Monocyte-derived DCs (MDDCs) from human blood and bone-marrow derived DCs (BMDCs) from 8–16-week-old male or female C57BL/6 mice (Zhang et al., 2012) were used in this study. MDDCs were generated as previously described (Borg et al., 2014), and cultured for 5 days in RPMI medium (BioWhittaker) supplemented with 10% fetal calf serum (FCS), 2 mM L-glutamine, 100 U/ml penicillin and 100 µg/ml streptomycin (Sigma-Aldrich). 100 ng/ml granulocyte-macrophage colony stimulating factor (GM-CSF; Immunotools) and 20 ng/ml IL-4 (Invitrogen, Life Technologies) were replenished every 2–3 days. BMDCs were cultured in medium supplemented with fetal calf serum and 50 ng/ml GM-CSF obtained from the supernatants of transfected J558 cells, as previously described (Faure-Andre et al., 2008). For the TFEB experiments, commercial GM-CSF (Sigma-Aldrich) was used. To induce DC maturation, the 2×10^6^ cells/ml were pulsed with 100 ng/ml LPS (Santa Cruz Biotechnology) for 30 min.

DCs and T cells from a healthy leukapheresis donor were used for the antigen presentation assay. Leukapheresis of a healthy donor was performed at the Department of Cellular Therapy, Radium Hospitalet, Oslo, Norway to harvest monocytes and lymphocytes. T cells from leukapheresis were thawed and expanded using Dynabeads CD3/CD28. In brief, T cells were cultured with Dynabeads (Dynabeads^®^*ClinExVivo*™ CD3/CD28, Thermo Fisher Scientific) at a 3:1 ratio in complete CellGro DC Medium (CellGenix) with 100 U/ml recombinant human IL-2 (Novartis) for 10 days. The T cells were frozen and aliquots were thawed and rested in complete medium [CellGro DC medium (CellGenix GmbH, Germany) supplemented with 5% heat-inactivated human pooled serum (TCS Biosciences Ltd, UK), 10 mM N-acetylcysteine (Mucomyst, 200 mg/ml; AstraZeneca AS, UK), 0.01 M HEPES (Life Technologies, Norway) and 0.05 mg/ml gentamycin (Garamycin; Schering-Plough Europe, Belgium)] before transfection and antigen presentation assay. Frozen monocytes were used to generate DCs for the antigen presentation assay. Briefly, monocytes were thawed and cultured 5 days in CellGro DC medium (CellGenix) supplemented with 100 ng/ml GM-CSF (Immunotools) and 20 ng/ml IL-4 (Invitrogen, Life Technologies) followed by 24 h maturation with LPS (100 ng/ml, Santa Cruz Biotechnology).

All experiments were performed in accordance with relevant guidelines and regulations. The animals were bred under conventional conditions, regularly screened for common pathogens and housed in compliance with guidelines set by the Experimental Animal Board under the Ministry of Agriculture of Norway. All experimental protocols involving transgenic and WT animals were approved by the National Committee for Animal Experiments (Oslo, Norway). Blood components (buffy-coats) from anonymous blood donors were obtained from the local blood bank (Section for Immunology and Blood Transfusion, Ullevål University Hospital, Oslo, Norway) according to the guidelines of the local blood bank approved by the Norwegian Regional Committee for Medical Research Ethics.

### Antibodies and reagents

The following antibodies were used for immunofluorescence (IF) and western blot (WB) experiments: anti-non-muscle myosin IIA (ab24762, abcam, IF 1:300), anti-myosin II light chain (M4401, Sigma-Aldrich, WB 1:200), anti-non muscle myosin IIA (ab55456, Abcam, IF 1:50), anti-phosphorylated myosin II light chain (#3671, Cell Signaling Technology, WB 1:500; IF 1:50), anti-vinculin (V9131, Sigma-Aldrich, IF 1:100), anti-tubulin (T9026, Sigma-Aldrich, WB 1:10,000), anti-TFEB (#4240, Cell Signalling Technology, WB 1:100), anti-Histone H3 (ab1791, Abcam, WB 1:100,000), anti-Rab7b (H00338382-M01, AbNova, WB 1:300; IF 1:20), anti-TRPML1 (Abcam, WB 1:100; IF 1:40), anti-TGN46 (AHP500, Bio-Rad, IF 1:250), anti-transferrin receptor (CBL47, Chemicon, IF 1:50), rabbit polyclonal antibody against LAMP2 (IF 1:1000; a gift from Sven Carlsson, University of Umeå, Sweden), anti-γ-tubulin (ab11316, Abcam, 1:200), and anti-α-tubulin (13-8000, Invitrogen, IF 1:200). Secondary antibodies conjugated to Alexa Fluor 488, Alexa Fluor 555 or Alexa Fluor 633 fluorophores (Life Technologies, 1:200) was used for immunofluorescence, while secondary antibodies conjugated with horseradish peroxidase (GE Healthcare, 1:500) were used for western blotting. Hoechst 33258 (H3569, Life Technologies) or DAPI (D9542, Sigma-Aldrich) was used at 0.2 µg/ml; Rhodamine-conjugated phalloidin (Invitrogen) was used at 33 nM; 10 kDa dextran, Alexa Fluor^®^ 647 conjugate (Life Technologies) was used at 120 µg/ml, and wheat germ agglutinin, Alexa Fluor^®^ 647 or 594 conjugate (WGA, Invitrogen) was used at 0.25 µg/ml. Fibronectin (Sigma-Aldrich) was used at 10 µg/ml for coating of coverslips. ML-SA1 (Sigma-Aldrich) was used overnight at a concentration of 20 µM.

The following antibodies were used for the flow cytometry experiments on human cells: HLA-DR (MHLDR05, Caltag Labs; 10 µl/test), CD80 (BD557227, BD Biosciences; 20 µl/test), HLA-ABC (BD562006, BD Biosciences; 20 µl/test), CCR7 (FAB197A, R&D Systems; 10 µl/test), CD11c (BD559877, BD Biosciences; 20 µl/test), CD83 (sc19678; 20 µl/test) and CD86 (sc19617; 20 µl/test) (both from Santa Cruz Technology). To check the expression of the TCR Radium-1 a Vβ3-FITC antibody was used (PN IM2372, Beckman Coulter-Immunotech; data not shown). As a degranulation marker for measuring antigen specific T cell activation upon antigen presentation from DCs, a CD107a–PE–Cy5 antibody was used (BD555802, BD Biosciences). CD8–PE–Cy7 (25-0088-42, Thermo Fisher Scientific) was used as a T cell marker to identify CD8+ cytotoxic T cells. Isotype controls IgG2a-FITC (sc2856), IgG1-FITC (sc2855), IgG1-PE (sc2866), IgG1-APC (sc2888) and IgG2b-APC (sc2890) (all from Santa Cruz Technology) were used. For Flow Cytometry on murine DCs, an antibody against CD86 (GL1, BD Biosciences) was used.

### RNA interference

For RNA interference (RNAi) in human DCs, the following siRNA oligonucleotides were used. For Rab7b siRNA, sense sequence 5′-GUAGCUCAAGGCUGGUGUATT-3′ and antisense sequence 5′-UACACCAGCCUUGAGCUACTT-3′. As negative control, we used the sense sequence: 5′-ACUUCGAGCGUGCAUGGCUTT-3′ and antisense control 5′-AGCCAUGCACGCUCGAAGUTT-3′. The oligonucleotides were purchased from Eurofins MWG Operon.

For RNAi in murine DCs, the following siRNA oligonucleotides were used. For Rab7b siRNA, sense sequence 5′-CAAUGGUAUCAACAUUCUATT-3′ and antisense sequence 5′-UAGAAUGUUGAUACCAUUGAG-3′. As negative control, we used the sense sequence: 5′-UUCUCCGAACGUGUCACGUTT-3′ and antisense sequence 5′-ACGUGACACGUUCGGAGAATT-3′. The murine oligonucleotides were purchased from Qiagen.

### Transfection by electroporation

After 5 days in culture, human DCs were collected, spun down at 300 ***g*** at 4°C, and washed in cold RPMI with no supplements. The cells were resuspended in cold RPMI to a concentration of 10^6^ cells per 300 µl, and electroporated with 100 nM siRNA in a 4 mm gap size cuvette (VWR). Electroporation was performed with an ECM 830 Square Wave Electroporation System (BTX Technologies Inc.,) for 3 ms at 500 V. Following electroporation, the cells were plated in complete RPMI medium and kept at 37°C with 5% CO_2_ for minimum 24 h before further experiments.

Expanded T cells were electroporated with mRNA encoding for Radium-1 TCR, specific for the tumor neoantigen encoded by the *TGFBR2* frameshift mutant, peptide_127-145_ KSLVRLSSCVPVALMSAMT (Inderberg et al., 2017). Briefly, T cells were washed twice and resuspended in cold RPMI medium to 20×10^6^ cells/ml. The mRNA was mixed with the cell suspension at 100 μg/ml, and electroporated in a 2 mm gap cuvette at 250 V and 2 ms using a BTX 830 Square Wave Electroporator (BTX Technologies Inc.). Immediately after transfection, T cells were transferred to complete culture medium [CellGro DC medium (CellGenix GmbH, Germany) supplemented with 5% heat-inactivated human pooled serum (TCS Biosciences Ltd, UK), 10 mM N-acetylcysteine (Mucomyst, 200 mg/ml; AstraZeneca AS, UK), 0.01M HEPES (Life Technologies, Norway) and 0.05 mg/ml gentamycin (Garamycin; Schering-Plough Europe, Belgium)] at 37°C in 5% CO_2_ overnight.

BMDCs were collected on day 6–7 of differentiation, and were transfected using the Amaza mouse Dendritic Cell Nucleofector Kit (Lonza), according to the manufacturer's specifications. Briefly, cells were resuspended to 5×10^6^ cells per 100 µl of Amaxa Solution containing 1 µM siRNA. After electroporation with an Amaxa Biosystems Nucleofector II electroporator (protocol Y-001), cells were incubated at 37°C for 30 min, before washing steps and re-plating for later experiments on day 10–12. The silencing efficiency was controlled either by western blotting or by quantitative real-time RT-PCR.

### Quantitative real-time RT-PCR

RNA extraction was undertaken by using an miRNeasy mini kit (Qiagen), following the manufacturer's protocol. cDNA was produced from 1 µg of RNA by using the SuperScriptVILO cDNA synthesis kit (Thermo Fisher Scientific). Quantitative PCR was performed to amplify and quantify cDNA using real-time reverse transcriptase (RT)-PCR with the Lightcycler 480 SYBR green I master mix and the Lightcycler 480 PCR system (Roche). Primers for murine *Rab7b* (forward primer, 5′-CGAGGAATACCAGACCACACT-3′; reverse primer, 5′-GGCTGGCCAGAACCTCAAAGG-3′, and for murine *Actb* (forward primer, 5′-AGTGTGACGTTGACATCCGT-3′; reverse primer, 5′-GCAGCTCAGTAACAGTCCGC-3′) were purchased from Eurofins MWG Operon. The PCR program was as follows: 1 cycle 3 min at 94°C; 40 cycles 15 s at 95°C, 30 s at 60°C, and 30 s at 72°C; 1 cycle 6 s at 75°C. The specificity and the identity of the PCR products were checked by a melting curve test. Actin transcript levels were used for the normalization of the samples.

### Western blotting

DC lysates were separated by SDS-PAGE, transferred onto a PVDF membrane (Millipore) and subjected to immunoblot analysis, with specific primary antibodies diluted in 2% non-fat dry milk (Bio-Rad) overnight at 4°C, followed by incubation with secondary HRP-conjugated antibodies for 1 h at room temperature. Bands were visualized by using the ECL system (GE Healthcare), and protein levels were quantified by densitometry using ImageQuant TL software (GE Healthcare).

### Antigen presentation assay

Radium-1 TCR-expressing T cells were stimulated for 5 h with DCs electroporated with either control siRNA or Rab7b siRNA and loaded with 10 µM of 19-mer peptide encoded by the *TGFBR2* frameshift KSLVRLSSCVPVALMSAMT (amino acid sequence 127–145; provided by Norsk Hydro ASA). The T-cell to target ratio was one T cell per two DCs, and the cells were incubated in the presence of an anti-CD107a antibody, as well as BD GolgiPlug (BD555029) and BD Golgistop (BD554724, both from BD Biosciences) at a 1:1000 dilution. Cells were then washed and surface stained with anti-CD8 antibody for flow cytometric analysis.

### Flow cytometry

Immature and activated DCs were harvested, washed three times with cold 1× PBS containing 0.05% BSA, and stained for flow cytometry on ice for 30 min with the indicated antibodies. After staining, the cells were washed three times with cold 1× PBS containing 0.05% BSA, before fixation in 3% PFA. The antigen presentation assay was performed on a BD FACSCanto II Flow Cytometer, while phenotype experiments were performed on a LSR II Flow Cytometer, and data was analyzed using FlowJo software (Tree Star Inc, USA).

### Immunofluorescence

MDDCs grown on poly-L-lysine coated coverslips (BioCoat) were gently washed with 1× PBS, then fixed in 3% PFA for 20 min at room temperature. Fixed samples were quenched for 10 min in NH_4_Cl 50 mM, and permeabilized with 0.25% saponin (Sigma-Aldrich) in PBS for 10 min, before staining with primary antibodies for 20 min, followed by 20 min incubation with Alexa-Fluor-conjugated secondary antibodies. Coverslips were mounted with Mowiol (Sigma-Aldrich), and confocal images were acquired on an Olympus FV1000 confocal scanning laser upright microscope (BX61WI) with a PlanApo 60×1.10 NA oil objective. For DCs migrating in microchannels, the PMDS was gently removed before permabilization and staining, and the dishes were filled with PBS before imaging. Confocal images were acquired on a Zeiss LSM880 Fast AiryScan confocal microscope with a C Plan Apo 63×/1.4A oil objective. For the staining of the MTOC, cells were fixed in 3% PFA for 5 min followed by ice-cold methanol for 5 min. The cells were permabilized and stained using a buffer containing 20 mM HEPES pH 7.5, 50 mM NaCl, 3 mM MgCl_2_ and 0.1% Triton X-100.

### Preparation of microchannels and speed quantification

Microchannels were prepared as previously described (Faure-Andre et al., 2008). Briefly, polydimethylsiloxane (PDMS) was added to prefabricated molds, before activation in a plasma cleaner for 30 s and attachment of the PDMS piece to a glass-bottomed dish. These were further incubated with 20 µg/ml fibronectin (Sigma-Aldrich) for 1 h, and rinsed with 1× PBS. For quantification of mean velocity and persistency, cells were loaded into microchannels and imaged for 20 h at 37°C with 5% CO_2_, on an epifluorescence Nikon TiE video-microscope equipped with a cooled CCD camera, using a 10× objective and acquiring one transmission phase image every 2 min. Extraction of kymographs and instantaneous velocity analysis were performed using an in-house MATLAB program as described previously (Faure-Andre et al., 2008). Graphs and statistical differences were assessed with Prism software, using a two-tailed unpaired Student's *t*-test.

### Migration in collagen gel

BMDCs were collected on day 9–10, stimulated with LPS for 30 min and replated in culture dishes. The following day, 7×10^4^ cells were resuspended in BMDC medium and mixed with 1.4 mg/ml collagen type I (Advanced BioMatrix) and 0.2% NaHCO_3_ (Sigma-Aldrich). From this, a 33.3 µl drop was spotted in a 24-well plate with glass bottom and topped with a coverslip to form a collagen sandwich. The plate was incubated at 37°C with 5% CO_2_ for 30 min to allow collagen polymerization. After this, 500 µl BMDC medium supplemented with 400 ng/ml chemokine [C-C motif ligand 21 (CCL21); BioLegend] was added to the wells as a chemoattractant. Cells were imaged every 2 min for 2 h using an Andor Dragonfly spinning disk microscope equipped with a CFI Plan Apo 10×/0.45 NA objective at 37°C with 5% CO_2_. Cells tracking was performed using the Manual Tracking plugin of ImageJ software (National Institutes of Health). Cell speed and directionality was determined using the Chemotaxis and Migration Tool software (Ibidi).

### Myosin and actin density map generation and analysis

Myosin and actin distribution analysis was performed on BMDCs loaded in 5×8 μm microchannels (4D Cell). Before seeding the cells, the channels were coated with 20 μg/ml of fibronectin as previously described. BMDCs were either plated untreated (for immature DCs) or treated with LPS to induce maturation for 30 min at 37°C, 5% CO_2_. 10×10^5^ cells were loaded into the access port for each channel and allowed to migrate overnight at 37°C, 5% CO_2_. On the following day, the cells in the channels were fixed with 3% PFA for 20 min and washed with PBS. After this, the PMDS structure on top of the channels was carefully removed before permeabilization with 0.25% saponin in PBS for 10 min followed by staining. All the steps were performed at room temperature. The cells were imaged using a Zeiss LSM880 Fast AiryScan confocal microscope with a C Plan Apo 63×/1.4A oil objective.

Density maps were generated using ImageJ. Briefly, images were cropped to contain single cells and resized to the average cell size. After background subtraction, the intensities were normalized and density maps were generated by projecting the mean signal of every individual cell and applying the physics look-up table (LUT; ImageJ) to the image. Intensities were measured in rectangles covering the front 20% of the cell, and in the back 20% of the cell.

Actin distribution analysis was performed similarly to the myosin distribution analysis. Rhodamine-conjugated phalloidin was used to stain the actin cytoskeleton and imaging was performed on an Andor Dragonfly microscope equipped with a CFI Plan Apo 100×/1.45 NA oil objective. Density maps were generated using the same method as for the myosin maps. Intensities were measured in a rectangle covering the first third of the cell, defined as the front, and in the last two thirds of the cell, defined as the back.

### Macropinocytosis and lysosome dynamics in migrating DCs

LPS-BMDCs were stained with WGA, Alexa Fluor^®^ 594 conjugate, as previously described (Bretou et al., 2017), before loading into 5×8 μm microchannels (4D Cell). The following day, channels were filled with 120 µg/ml 10 kDa Alexa Fluor 647-conjugated dextran (Life Technologies) for 30 min and then imaged every minute for 20 min using on an Andor Dragonfly microscope equipped with a CFI Plan Apo 100×/1.45 NA oil objective. A confocal section of the middle plane was selected, and multi-position mode was used to image multiple cells simultaneously from the same starting point. The area of internalized dextran and number of macropinosomes was quantified by drawing a region of interest (ROI) around each cell and using particle analysis in ImageJ to identify dextran-positive objects. Prior particle analysis, the images were processed with a median filter, and a threshold was applied to generate a binary image. The watershed function was used to split touching objects. The lysosomes were analyzed similarly, using particle analysis to identify WGA-positive objects. Macropinosome lifetime was estimated by counting the number of frames in which the same macropinosome was present.

### TFEB nuclear translocation assay

On day 7 of culture, BMDCs were treated with 100 ng/ml LPS for 30 min and lysed after 6 h with a cold lysis buffer containing 20 mM Tris-HCl pH 7.5, 150 mM NaCl (Sigma-Aldrich), 2 mM EDTA (VWR), 0.1% IGEPAL CA-630 (Sigma-Aldrich), protease and phosphatase inhibitors (Sigma-Aldrich). The samples were spun at 13,400 ***g*** for 1 min and the supernatant was collected as the cytosolic fraction. The pellet containing the nuclei was washed three times with cold lysis buffer. Both cytosolic and nuclear fractions were resuspended in Laemmli sample buffer containing 100 mM DTT. Cytosolic fractions were heated at 96°C for 5 min while nuclear fractions were heated at 96°C for 20 min. The denatured fractions were subjected to SDS-PAGE and analyzed by western blotting.

### Expression of His-tagged proteins and pull down

His-tagged Rab7bQ67L and His-tagged Rab33bQ92L were expressed in *Escherichia coli* BL21 (DE3; Agilent Technologies) transformed with pET16b His-Rab7bQ67L (Progida et al., 2012) and pET16b His-Rab3bQ92L (GenScript), after induction with 0.5 mM IPTG for 3 h at 37°C. The bacteria were centrifuged at 3000 ***g*** for 25 min, resuspended in 64 mM Tris-HCl pH 8.5, 8 mM MgCl_2_, 20 mM β-mercaptoethanol and 0.3 mM PMSF, and lysed with a French press. Expressed His-tagged proteins were purified from the bacterial soluble fraction using nickel-nitrilotriacetic acid resin (Qiagen) in the presence of 50 mM Tris-HCl pH 8.0, 500 mM NaCl, 5% glycerol, 1% Triton X-100, 5 mM β-mercaptoethanol, 20 mM imidazole and 0.3 mM PMSF, according to the manufacturer's protocol. For pulldown experiments, 40 µg of His–Rab fusion proteins bound to Dynabeads™ His-Tag were incubated with precleared lysates from LPS-treated MDDCs for 30 min at 4°C and then washed six times with buffer containing 3.25 mM sodium phosphate, pH 7.4, 70 mM NaCl and 0.01% Tween-20. Bound proteins were eluted with elution buffer (50 mM sodium phosphate, pH 8.0, 300 mM NaCl, 0.01% Tween-20, 300 mM imidazole). Samples were analyzed by using SDS-PAGE and immunoblotting.

### Image analysis, processing and statistical analysis

Images were processed with ImageJ and Adobe Photoshop (Adobe Systems Inc., CA, USA). Quantifications were undertaken using Fluoview 1000 (Olympus, Hamburg, Germany) and ImageJ. Statistical differences, unless otherwise stated, were assessed by two-tailed unpaired Student's *t*-test (Excel software). In the figures, statistical significance is indicated as follows: *P<0.05, **P<0.01, ***P<0.001.

## Supplementary Material

Supplementary information

Reviewer comments
