# Supplementary material for: Rab7b regulates dendritic cell migration by linking lysosomes to the actomyosin cytoskeleton
Source: J Cell Sci. 2021 Sep 24;134(18):jcs259221. doi: 10.1242/jcs.259221 (PMC8487646; doi:10.1242/jcs.259221)
Supplement: Supplementary information [file joces-134-259221-s1.pdf]

Sup.Fig.1

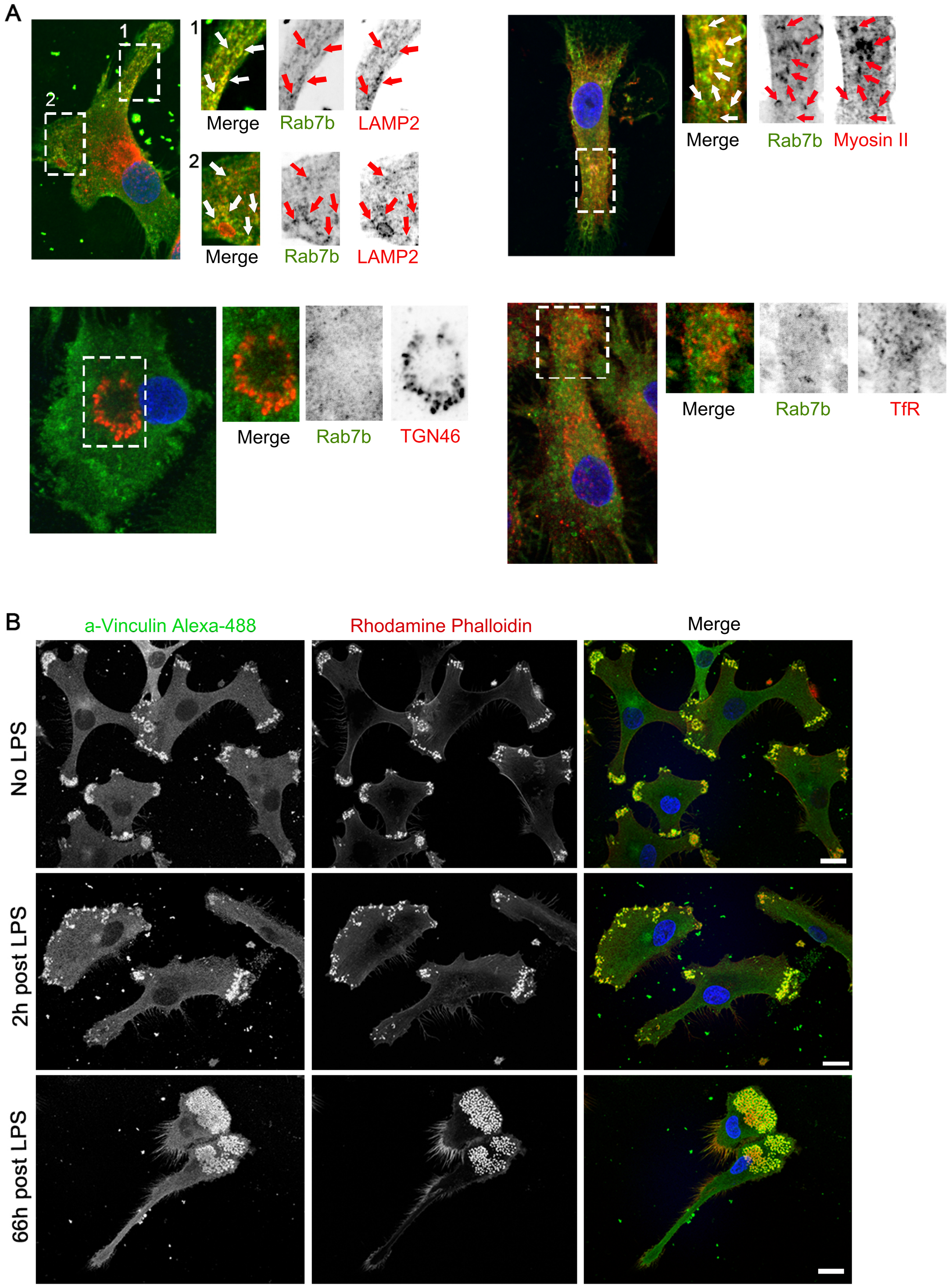

Journal of Cell Science • Supplementary information

Fig. 1. Localization of endogenous Rab7b and polarization of podosomes in mature DCs (**A**) MDDCs stimulated with LPS were plated on PLL-coated coverslips, fixed and immunostained with the indicated antibodies. Scale bar 10µm. (**B**) Immature MDDCs were plated on PLL-coated coverslips, and stimulated with 100 ng/ml LPS for either 2h or 66h before fixation and immunostaining with an antibody against vinculin (green). Actin was labeled with rhodamine-conjugated phalloidin (red) and nuclei with Hoechst (blue). Scale bar 10µm.

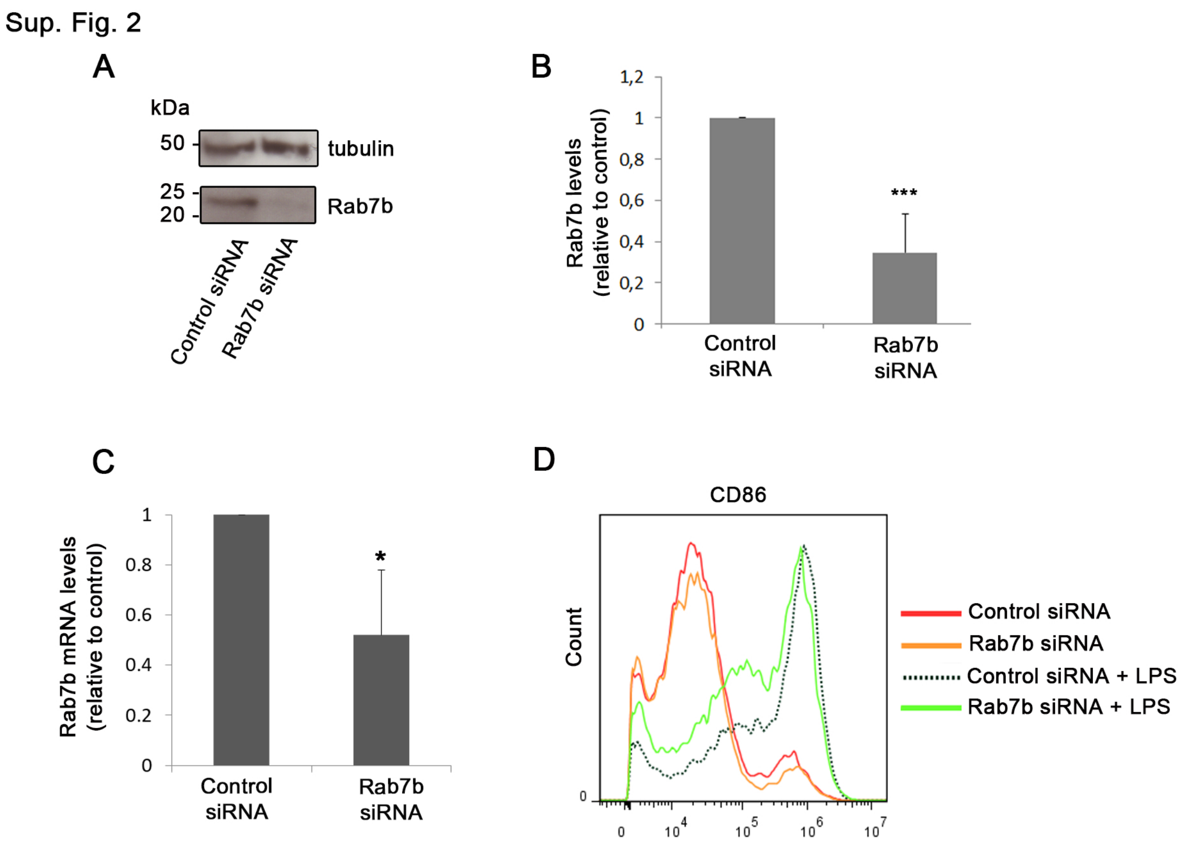

Fig. S2. Rab7b depletion in DCs

(A) DCs from a healthy donor were transfected by electroporation with either control siRNA or Rab7b siRNA, and subjected to Western blot analysis with antibodies against Rab7b and tubulin as a loading control. **(B)** Quantification of Rab7b levels in DCs silenced with control siRNA or Rab7b siRNA. The intensity of the bands from western blots was quantified using ImageQuant, and the level of Rab7b was normalized to the amount of tubulin. Data represents the mean ± s.d. of three independent experiments. ***$P < 0.0001$. **(C)** Quantification of Rab7b mRNA levels in BMDCs. Rab7b mRNA levels were quantified by real-time RT-PCR in BMDCs transfected with either control siRNA or Rab7b siRNA. The levels of Rab7b mRNAs were normalized to the amount of actin. Data represents the average of three independent experiments ± s.d. *$P < 0.05$. **(D)** FACS analysis of the surface expression marker CD86 in BMDCs. Data shows a representative histogram overlay in which the dotted and the red lines represent the control siRNA with and without LPS, while the green and the orange lines represent the Rab7b siRNA with and without LPS, respectively.

Journal of Cell Science • Supplementary information

Supplementary Figure 3

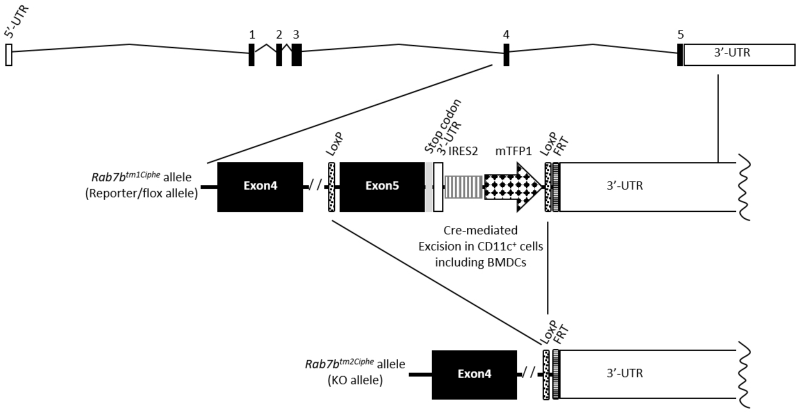

Fig. S3. Schematic representation of the genetic engineering of the *Rab7b* alleles

A LoxP site was introduced 5' of exon 5 and an IRES2-mTFP1-LoxP cassette was inserted downstream of the stop codon in the 3′ -untranslated region of exon 5 of the *Rab7b* gene, by classical homologous recombination in the C57BL6/N EUCOMM JM8.F6 ES cell line, generating the *Rab7b*^{tm1Ciphe} allele preserving the expression of the *Rab7b* gene but reporting it via the expression of the mTFP1 fluorescent protein and enabling its knock-out upon deletion of exon 5 together with the reporter cassette after crossing to Cre-expressing mice. The *Rab7b*^{tm2Ciphe} conditional knock-out allele was generated specifically in CD11c+ cells, including BMDCs, by crossing mice bearing the *Rab7b*^{tm1Ciphe} allele with CD11c-Cre transgenic mice (B6.Cg-Tg(Itgax-cre)1-1Reiz/J).

Journal of Cell Science • Supplementary information

Supplementary Figure 4

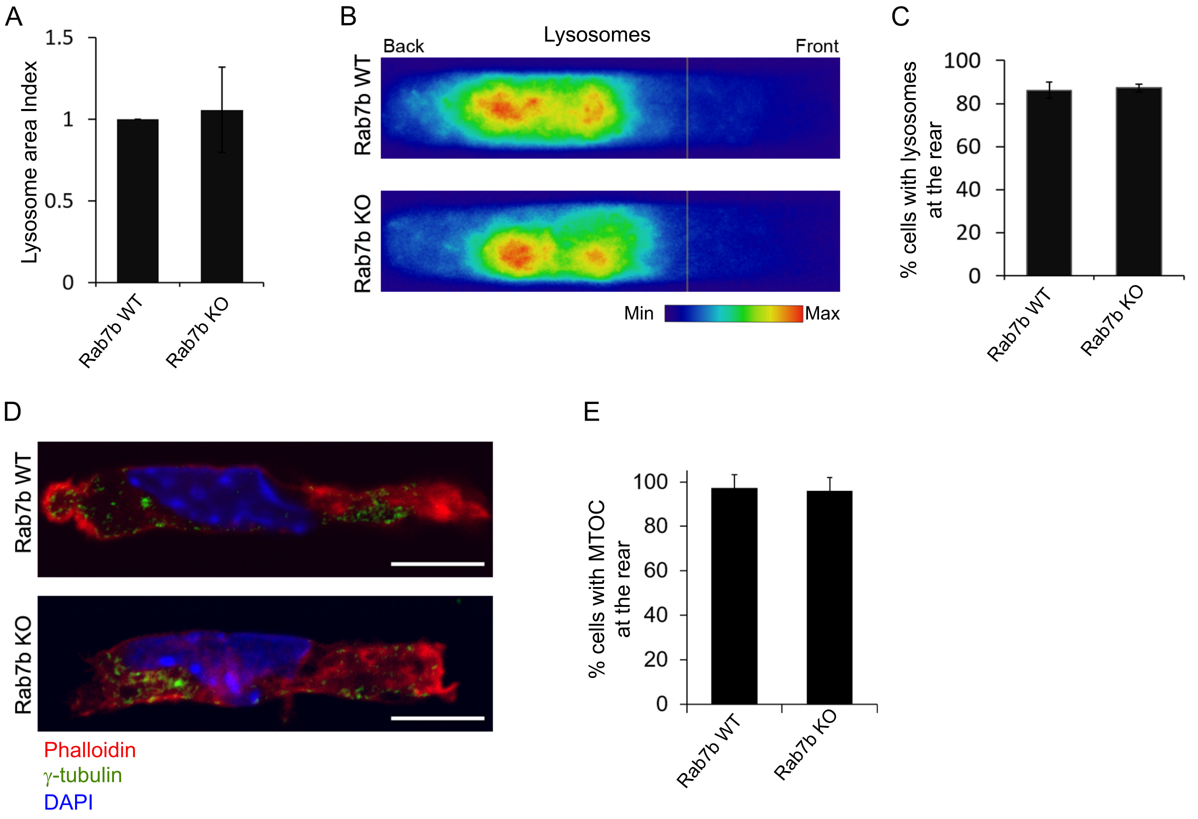

Fig. S4. Rab7b does not affect lysosome or MTOC orientation

(A-C) WT and Rab7b KO DCs were incubated with AF647-WGA to label lysosomes, treated with LPS, loaded in 5 x 8 µm micro-fabricated channels and fixed after 16 hours. **(A)** Quantification of the lysosome area index, defined as the area of the lysosomes relative to size and normalized to control. **(B)** The intensity of each cell for each condition was averaged into a single density map. One representative experiment out of three is shown. **(C)** Quantification of the percentage of cells with the main lysosome cluster localized at the cell rear (defined as last two thirds of the cell). Data represents the average of three independent experiments ± s.d. (n=51 and 57 cells for WT and Rab7b-KO, respectively). **(D)** LPSDCs were loaded in 5 x 5-8 µm wide micro-fabricated channels, fixed after 16 hours and stained with an antibody against -tubulin to label the MTOC. Rhodamine-conjugated phalloidin was used to visualize actin and DAPI to label the nucleus. Representative images from one out of three independent experiments are shown. Scale bar 10 µm. **(E)** Quantification of the percentage of cells with the MTOC positioned at the rear. Data represents the average of three independent experiments ± s.d. (n=30 and 35 cells for WT and Rab7b-KO, respectively).

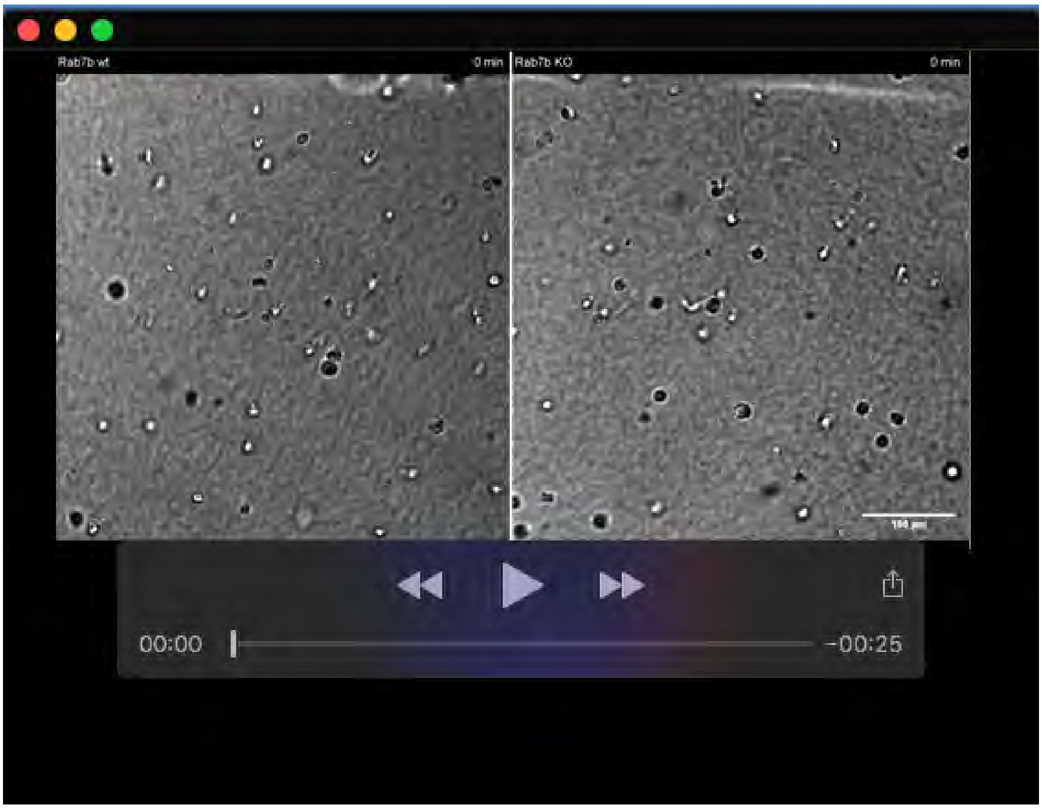

Movie 1. WT and Rab7b-KO DCs migrating in collagen gels

WT and Rab7b-KO LPS-DCs migrating in collagen gels. The source of the CCL21 chemoattractant is at the top of the movie. Cells were imaged every two minutes for two hours. Scale bar: 100 µm.

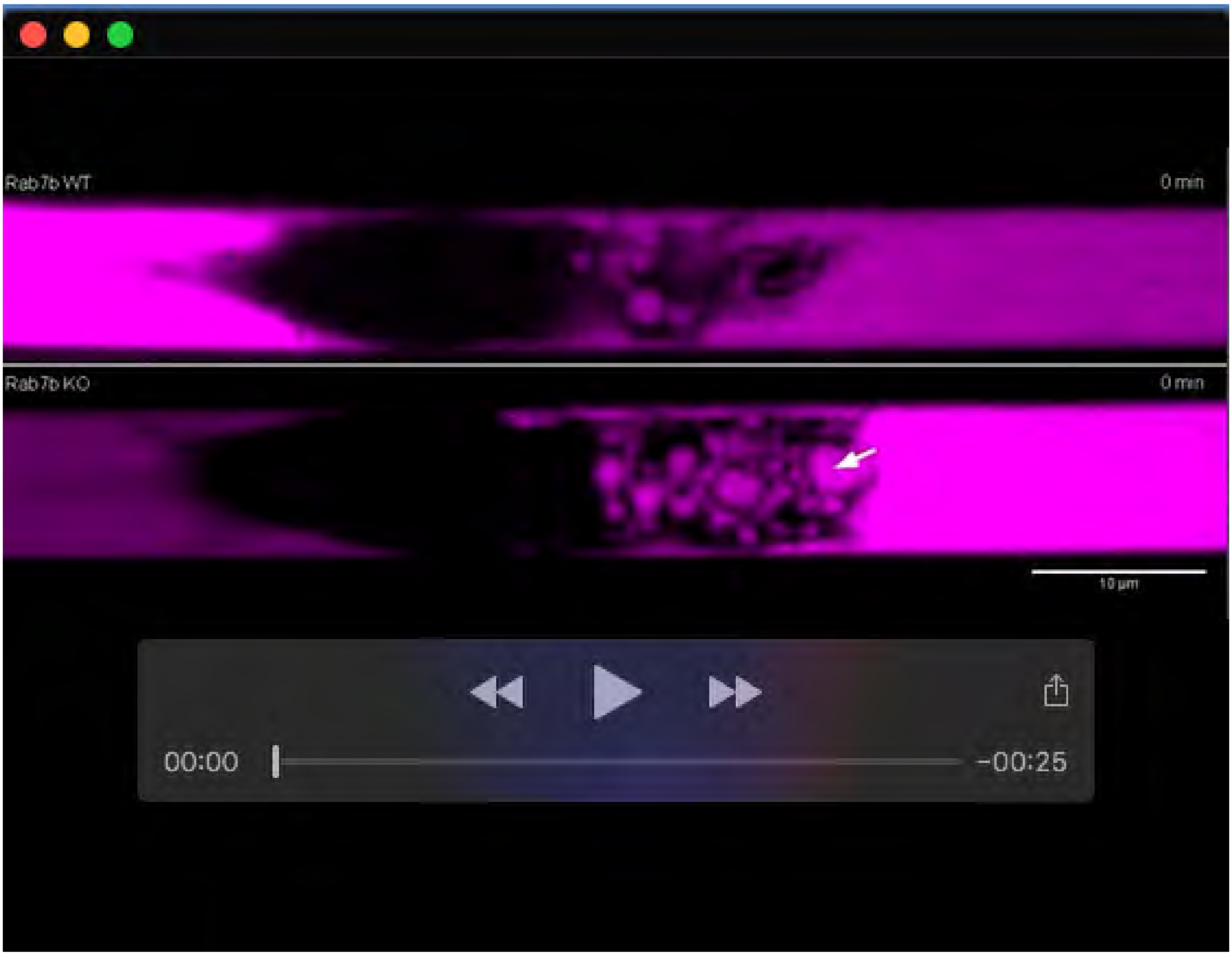

Movie 2. Macropinocytosis in WT and Rab7b-KO DCs
WT and Rab7b-KO LPS-DCs were loaded in 5 x 8 µm micro-fabricated channels. After 16 hours, the channels were filled with 10 kDa 647-Dextran (magenta), and 30 minutes later the cells were imaged at imaged at 1-minute intervals. The arrow shows an example of a macropinosome with long liftetime in the Rab7b-KO cell. Scale bar: 10 µm.

Journal of Cell Science • Supplementary information

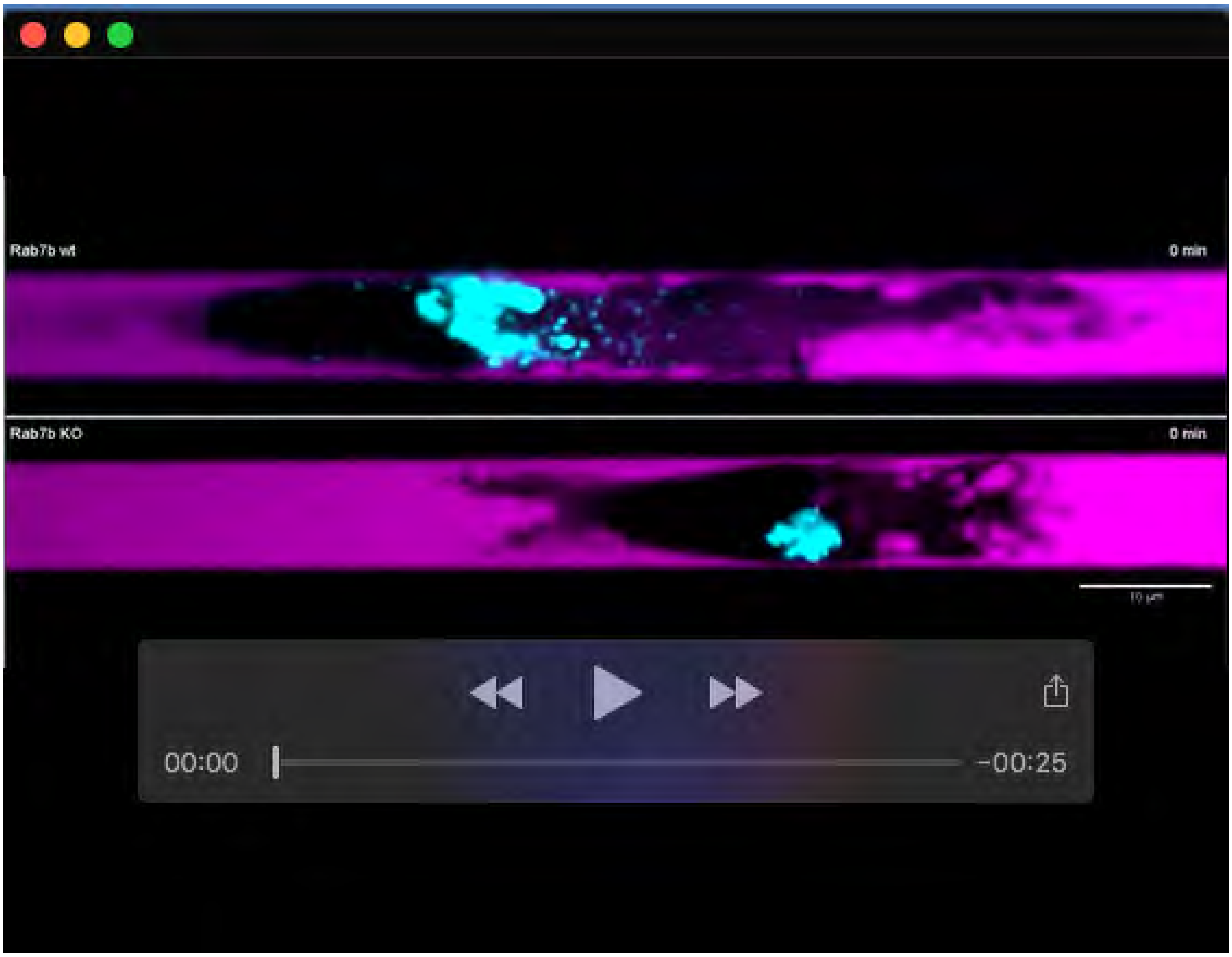

Movie 3. Lysosome dynamics during macropinocytosis in WT and Rab7b-KO DCs
WT and Rab7b-KO LPS-DCs stained with AF594-WGA to label lysosomes (cyan) were loaded in 5 x 8 µm micro-fabricated channels. After 16 hours, the channels were filled with 10 kDa 647-Dextran (magenta), and 30 minutes later the cells were imaged at imaged at 1-minute intervals. Scale bar: 10 µm.

Journal of Cell Science • Supplementary information
