# Supplementary material for: Rab7b regulates dendritic cell migration by linking lysosomes to the actomyosin cytoskeleton
Source: J Cell Sci. 2021 Sep 24;134(18):jcs259221. doi: 10.1242/jcs.259221 (PMC8487646; doi:10.1242/jcs.259221)
Supplement: Reviewer comments [file joces-134-259221_review_history.pdf]

Rab7b regulates dendritic cell migration by linking lysosomes to the actomyosin cytoskeleton

Katharina Vestre, Irene Persiconi, Marita Borg Distefano, Nadia Mensali, Noemi Antonella Guadagno, Marine Bretou, Sébastien Wälchli, Catharina Arnold-Schrauf, Oddmund Bakke, Marc Dalod, Ana-Maria Lennon-Dumenil and Cinzia Progida
DOI: 10.1242/jcs.259221

Editor: Daniel Billadeau

Review timeline

Submission to Review Commons:	22 January 2021
Submission to Journal of Cell Science:	2 August 2021
Editorial decision:	16 August 2021

Reviewer 1

Evidence, reproducibility and clarity

In the present manuscript Vestre and colleagues describe the role of the small GTPase Rab7b during immune cell migration using dendritic cells as a prototypic cell for amoeboid migration. Rapid and persistent dendritic cell migration is regulated by lysosomal signaling, which in turn promotes actomyosin activation at the cell's uropod. The authors demonstrate that depletion of Rab7b in dendritic cells impairs cell polarization and directional migration in linear microfabricated channels (1D) as well as in 3D collagen matrices. Analysis of actin distribution in mature dendritic cells revealed higher concentrations at the cell front and sustained macropinocytic activity upon Rab7b depletion while lysosome numbers and lysosome-associated signaling were decreased. Similarly, myosin II phosphorylation was diminished in Rab7b-depleted dendritic cells. Altogether, the authors identify Rab7b as the mechanistic link, which mediates between lysosomal signaling and activation of the actomyosin cytoskeleton thus ensuring directional locomotion.

Major comments:

This work nicely addresses the function of Rab7b in dendritic cells. The manuscript is well written and easy to follow. The experiments are technically well performed and well- presented and generally support the authors conclusions. Solely one point could strengthen the authors conclusions concerning the interaction between Rab7b and myosin:

The authors demonstrate that Rab7b-depletion functionally impairs dendritic cells migration (both cell speed and persistence). They link altered actin localization and lysosomal signaling in Rab7b depleted cells to the migratory phenotype observed in these cells. While they clearly demonstrate that actin is shifted to the cell's front (Figure 4A-C) it is less clear what happens to myosin II (Figure 5). Earlier studies show that upon antigen encounter lysosomal calcium is released by the ionic channel TRPML1, which activates myosin II at the cell rear (Bretou et al., 2017).
Here it would be nice if the authors could show how myosin II localizes in Rab7b-deficient cells and whether myosin phosphorylation is affected at the cell rear to strengthen their hypothesis that Rab7b is involved in the recruitment of myosin II to lysosomes.

Some of their statistical tests are not clear: why are the authors using paired t-tests in Figure 3G and 5G? I do not understand why the data set is paired here?

© 2021. Published by The Company of Biologists under the terms of the Creative Commons Attribution License (https://creativecommons.org/licenses/by/4.0/).

Minor comment:

I would suggest to display all data as mean {plus minus} standard deviation (sd) instead of standard error of the mean (sem).

Significance

Overall, the story nicely advances previous studies showing a direct interaction between Rab7b and myosin II (Borg et al., 2014; Distefano et al., 2015) and lysosomal signaling being required for fast and efficient dendritic cell trafficking (Bretou et al., 2017). The present study now addresses the mechanistic link between lysosomes and the actomyosin cytoskeleton, which to date is ill defined. The findings will be of great interest for the field of cell biology as well as immunology as they contribute to a better molecular understanding of amoeboid migration strategies - the preferred migratory mode of immune cells.

Reviewer 2

Evidence, reproducibility and clarity

The work by Vestre et al., is a broad study focused on the effect of GTPase Rab7b deficiency in dendritic cells, including the description of cell morphology and distribution of F-actin-based structures during migration. Authors find that cell directionality and speed decrease during migration when Rab 7b is absent, and describe a specific phenotype in the organization of lysosomes, including a differential nuclear/cytosol distribution of TFEB. They also observe that the organization and activity of Myosin IIA (by means of phosphorylation of myosin light chain) is disturbed in Rab7b deficient cells.

There is no mechanistic insight explaining the effects observed by the lack of Rab7b, although correlation between myosin IIA, F-actin and lysosome distribution and organization is described. Authors have not addressed the endogenous localization of Rab7b in their cellular system. Does Rab7b localize to lysosomes, to endosomes, to the trans-Golgi compartment? Is there any change in the localization and structure of these compartments when cells polarize upon LPS stimulus? Specific immune-localization of endogenous protein is required to assess this issue.

More specific markers for lysosomes should be added for the cell localization studies. Do lysosomes localize to the rear edge of polarized dendritic cells? Are they bound to microtubules or to F-actin? Are microtubules and centrosome correctly located for migration in the Rab7b silenced cells?

Where is myosin light chain phosphorylated in DCs? Localization of myosin light chain(phosphorylated) should be assessed in LPS-matured DC in the presence and the absence of Rab7b. Does this phosphorylation increase with LPS and polarization? Is this affected by Rab7b silencing and therefore may have a role in DC migration?

Significance

This an interesting study, but the findings described are not well connected. It needs additional experimentation to provide a more mechanistic view.

Reviewer 3

Evidence, reproducibility and clarity

Vestre et al investigate the consequences of the loss of the small GTPase Rab7b on DC migration, using monocyte-derived human dendritic cells (MDDCs) and bone-marrow derived mouse dendritic cells (BMDCs).

They reveal that loss of Rab7b results in (1) a (mild; 20% speed reduction) defect in DC velocity, (2) a defect in DC polarity, (3) an increased macropincocytosis-like vesicular uptake, (4) changes in

© 2021. Published by The Company of Biologists under the terms of the Creative Commons Attribution License (https://creativecommons.org/licenses/by/4.0/).

lysosomal dynamics, (5) reduced nuclear translocation of the lysosome regulator (transcription factor) TFEB, and (6) reduced MLC phosphorylation. The authors convincingly exclude that these effects are a consequence of a defective DC maturation phenotype.

The results are interesting, and appear clear and reproducible; especially the DC migration/polarisation phenotype is reproducible by alternative loss-of-function approaches (knockdown and knockout) and alternative migration assays (3D collagen matrices and microchannels). However, the causality between some of the different findings and thus parts of the proposed mechanisms are not entirely clear.

Major Point:

A number of causality arguments in the manuscript build on previous findings from below mentioned manuscripts (see 'significance') and from Vargas at al 2016 (Bretou, M. et al. Lysosome signaling controls the migration of dendritic cells. Science Immunology 2, eaak9573 (2017); Vargas, P. et al. Innate control of actin nucleation determines two distinct migration behaviours in dendritic cells. Nature cell biology 18, 43-53 (2016).). Yet, the direct causality regarding the different identified Rab7b loss-of-function phenotypes should be clarified by Vestre et al, or at least the causality claims in the manuscript should be changed. The authors write (e.g. abstract line 40) that they identify Rab7b as the missing physical link between lysosomes and the actomyosin cytoskeleton. However the manuscript does not include experiments directly showing this physical link, and also not indirectly (e.g. myosin localisation upon Rab7b depletion; Rab7b localisation). Given the observed defects in nuclear translocation of the transcription factor TFEB in Rab7b depletion DCs, one could speculate that the findings could also be explained by changes in transcriptional regulation (instead of a physical linkage). Further, the causality between enhanced macropinocytosis phenotype and reduced cell migration phenotype is not directly shown, as the authors currently only correlate both findings with each other: could the authors perform experiments to directly address the causality that the enhanced macropinocytosis rate in Rab7b KD/KO cells indeed is causative for the reduced migration speed (e.g. does macropinocytosis inhibition in Rab7b KOs rescue the cell migration phenotype)?

Further Points:

- Line 34: The statement "Rab7b is a key regulator of migration of DCs..." appears exaggerated given the approx. 20% speed reduction of Rab7b KO/KDs.
- Timing of Rab7b up regulation: the authors state that their previously identified up regulation of Rab7b correlates with an enhanced DC migratory ability (e.g. lines 270-274). Yet, in their previous manuscript the authors showed an early up regulation of Rab7b on RNA and protein level 4 hours after LPS stimulation, a time point where the macropinocytosis activity might rather be high and the migratory activity rather low. At later time points, the RNA and protein levels drop to the original level again, and for the protein level even lower than the unstimulated control. Yet, these are the timepoints where the authors e.g. investigate DC migration/macropinocytosis in the microchannels (up to 20 hours). The authors should clarify this aspect and explain how this aspect fits into their model.
- Podosomes are adhesive structures rather observed in immature DCs than mature DCs. Yet, the authors identify a reduced migration speed only in mature and not immature DCs. Thus, it is unclear why the authors investigate the polarity of the actin cytoskeleton in mature DCs on highly adhesive PLL-coated coverslips (potentially artificially inducing podosome formation). More appropriate could be the use of less-adhesive assays, such as the 'PDMS- confiner' (Maiuri, P. et al. Actin flows mediate a universal coupling between cell speed and cell persistence. Cell 161, 374-386 (2015).) or the under-agarose assay that is more simple to establish in every laboratory (Heit, B. & Kubes, P. Measuring Chemotaxis and Chemokinesis: The Under-Agarose Cell Migration Assay. Sci. Signal. 2003, pl5-pl5 (2003).).
- Line 151: It is written 'Rab7b KO DCs', however, the corresponding figure indicates that Rab7b-depleted (and not knockout) cells have been used in this experiments. These authors should check whether the experimental details on knockout versus knockdown have been accurately described in all figures, legends, and text paragraphs.
- Line 174 and corresponding Figure: it is stated that the fraction of actin located at the cell front is 30% higher in Rab7b KO DCs. However, the data in Fig. 4B rather show an equal actin signal in

© 2021. Published by The Company of Biologists under the terms of the Creative Commons Attribution License (https://creativecommons.org/licenses/by/4.0/).

front; the ratio seems to be only changed due to a decreased actin signal in the cell back. The authors should clarify this aspect and explain how this finding fits into their model.
- The authors use the distribution of actin and the vesicle volume as a read out for the efficiency of macropinocytosis. Macropinocytosis research typically takes advantage of large labeled dextran (70kDa) to specifically investigate macropinocytosis (e.g. Commisso C, Flinn R J and Bar-Sagi D 2014 Determining the macropinocytic index of cells through a quantitative image-based assay. Nature Protocols 9 182-92). Thus, it is surprising that the authors used a 10kDa dextran that could also be taken up by other endocytotic pathways. Moreover, the authors could control whether the indeed observe macropinocytosis by using a macropinocytosis inhibitor like EIPA. Further, it is surprising to see that the intra-vesicular dextran intensity appears equal in the depicted images, yet, a characteristic of macropinocytosis-vesicles is their maturation that goes in hand with volume shrinkage and thus fluid-concentration (increase in dextran intensity; e.g. Freeman S A, Uderhardt S, Saric A, Collins R F, Buckley C M, Mylvaganam S, Boroumand P, Plumb J, Germain R N, Ren D and Grinstein S 2020 Lipid-gated monovalent ion fluxes regulate endocytic traffic and support immune surveillance Science 367 301-5)
- Figure 4D and E: The quantification in E shows an approx. doubling in vesicle area in Rab7b KOs, yet the image in D shows a much extremer difference. It would be recommended to use a representative image for Figure 4D.
- Figure 4G: According to the methods section, this analysis has been performed with images in 1min intervals. Thus, it appears rather inappropriate to show a scale in seconds; given the small in difference of roughly 20sec, it is unclear whether this experimental setup can really infer the conclusion (longer vesicle lifetime).
- Figure 5A/B: the graph in B only quantifies the number of small lysosomes, finding a reduction in Rab7b KO DCs. However, the picture in A indicates that there is actually an increase in overall lysosome staining in Rab7b KO DCs. It is unclear why the authors do not quantify and comment on this aspect.
- Statistics: the authors frequently show s.e.m.; thereby, however, error bars get smaller with higher n numbers. To exclude this effect in datasets with high n numbers, it is recommended to depict e.g. the 95% confidence interval (CI; e.g. Cumming, G., Fidler, F. & Vaux, D. L. Error bars in experimental biology. J Cell Biology 177, 7-11 (2007).). Moreover, it is unclear why some graphs do not show any SD/SEM/CI (E.g. Fig 1C, 3C, 3D, 3F). Further, it would be helpful to plot individual datapoints into the bar graphs to be able to judge the spread of the data.

Significance

Dendritic cells (DCs) link the innate and adaptive immune system by their ability to migrate from peripheral tissue locations (e.g. skin) to secondary lymphoid organs (lymph nodes). Whereas several regulators of DC migration have been already identified (e.g. actin cytoskeletal regulators) the field clearly lacks an understanding of additional molecular pathways responsible for DCs migration. In the accompanying manuscript, Vestre et al investigate how the loss of the small GTPase Rab7b affects DC migration in vitro.

In previous papers, the authors already showed that

1) Rab7b interacts with myosin II in human DCs (using immunopreciptation and co- localisation) and alters actin stress fibre formation, myosin light chain (MLC) phosphorylation, cell spreading and cell migration in HeLa cells (Borg, M., Bakke, O. & Progida, C. A novel interaction between Rab7b and actomyosin reveals a dual role in intracellular transport and cell migration. J Cell Sci 127, 4927-4939 (2014).)

2) Rab7b is up up-regulated on RNA and protein level in DCs upon LPS simulation (Berg- Larsen, A., Landsverk, O. J. B., Progida, C., Gregers, T. F. & Bakke, O. Differential Regulation of Rab GTPase Expression in Monocyte-Derived Dendritic Cells upon Lipopolysaccharide Activation: A Correlation to Maturation-Dependent Functional Properties. Plos One 8, e73538 (2013).).

Building on these results, Vestre et al investigate now the consequences of the loss of the small GTPase Rab7b on DC migration.

© 2021. Published by The Company of Biologists under the terms of the Creative Commons Attribution License (https://creativecommons.org/licenses/by/4.0/).

Author response to reviewers' comments

Reviewer #1 (Evidence, reproducibility and clarity (Required)):
In the present manuscript Vestre and colleagues describe the role of the small GTPase Rab7b during immune cell migration using dendritic cells as a prototypic cell for amoeboid migration. Rapid and persistent dendritic cell migration is regulated by lysosomal signaling, which in turn promotes actomyosin activation at the cell's uropod.
The authors demonstrate that depletion of Rab7b in dendritic cells impairs cell polarization and directional migration in linear microfabricated channels (1D) as well as in 3D collagen matrices. Analysis of actin distribution in mature dendritic cells revealed higher concentrations at the cell front and sustained macropinocytic activity upon Rab7b depletion while lysosome numbers and lysosome- associated signaling were decreased. Similarly, myosin II phosphorylation was diminished in Rab7b- depleted dendritic cells. Altogether, the authors identify Rab7b as the mechanistic link, which mediates between lysosomal signaling and activation of the actomyosin cytoskeleton thus ensurig directional locomotion.

Major comments:

This work nicely addresses the function of Rab7b in dendritic cells. The manuscript is well written and easy to follow. The experiments are technically well performed and well-presented and generally support the authors conclusions. Solely one point could strengthen the authors conclusions concerning the interaction between Rab7b and myosin:
The authors demonstrate that Rab7b-depletion functionally impairs dendritic cells migration (both cell speed and persistence). They link altered actin localization and lysosomal signaling in Rab7b depleted cells to the migratory phenotype observed in these cells. While they clearly demonstrate that actin is shifted to the cell's front (Figure 4A-C) it is less clear what happens to myosin II (Figure 5). Earlier studies show that upon antigen encounter lysosomal calcium is released by the ionic channel TRPML1, which activates myosin II at the cell rear (Bretou et al., 2017). Here it would be nice if the authors could show how myosin II localizes in Rab7b-deficient cells and whether myosin phosphorylation is affected at the cell rear to strengthen their hypothesis that Rab7b is involved in the recruitment of myosin II to lysosomes.

We thank the reviewer for the positive evaluation of our work. We have now performed the suggested experiments and found that the front/back ratio of myosin II in Rab7b-deficient dendritic cells migrating in microchannels is indeed 32% higher than in Rab7b WT cells, indicating that Rab7b KO cells fail to recruit myosin II to the cell rear after addition of LPS (Figure 4A-C). Also, myosin phosphorylation at the cell rear is reduced in Rab7b KO dendritic cells as shown in figure 6C-D. We further demonstrated by pulldown experiments that Rab7b interacts with the lysosomal calcium channel TRPML1 (Figure 6G). This, together with the immunofluorescence analysis showing colocalization between Rab7b and TRPML1 (Figure 6H) strengthens the hypothesis that Rab7b recruits myosin II to lysosomes, bringing the motor in close proximity to TRPML1 which activates myosin II at the cell rear.

Some of their statistical tests are not clear: why are the authors using paired t-tests in Figure 3G and 5G? I do not understand why the data set is paired here?

We assume that the reviewer refers to figures 3F and 4G (Fig. 5D in the revised manuscript), where we have used paired t-tests. We used the paired t-test in figure 3F (Quantification of the mean cell speed of WT and Rab7b KO LPS-DCs) as we noticed a small variability in the cell speed between the different experiments dependent on the technical variability of the collagen gel preparation. However, the relative difference between WT and KO dendritic cells remained similar. As in each experiment, WT and KO dendritic cells were embedded in collagen gels prepared on the same day (from the same master mix), we considered the samples run on the same collagen preparation as matched pair to account for the dependency between cell speed and collagen preparation.

Similarly, we considered the data set in figure 5D as matched pair, because the time interval between frames in the last experiment was slightly different compared to the first two experiments due a technical problem with the microscope. However, we have now changed this graph and presented the data relative to control to account for a comment by reviewer 3. Therefore, in the new graph we have used the unpaired t test.

© 2021. Published by The Company of Biologists under the terms of the Creative Commons Attribution License (https://creativecommons.org/licenses/by/4.0/).

Minor comment:
I would suggest to display all data as mean ± standard deviation (sd) instead of standard error of the mean (sem).

We have replaced the standard error of the mean with standard deviation according to the reviewer's suggestion.

Reviewer #1 (Significance (Required)):
Overall, the story nicely advances previous studies showing a direct interaction between Rab7b and myosin II (Borg et al., 2014; Distefano et al., 2015) and lysosomal signaling being required for fast and efficient dendritic cell trafficking (Bretou et al., 2017). The present study now addresses the mechanistic link between lysosomes and the actomyosin cytoskeleton, which to date is ill defined. The findings will be of great interest for the field of cell biology as well as immunology as they contribute to a better molecular understanding of amoeboid migration strategies - the preferred migratory mode of immune cells.

Reviewer #2 (Evidence, reproducibility and clarity (Required)):
The work by Vestre et al., is a broad study focused on the effect of GTPase Rab7b deficiency in dendritic cells, including the description of cell morphology and distribution of F-actin-based structures during migration. Authors find that cell directionality and speed decrease during migration when Rab 7b is absent, and describe a specific phenotype in the organization of lysosomes, including a differential nuclear/cytosol distribution of TFEB. They also observe that the organization and activity of Myosin IIA (by means of phosphorylation of myosin light chain) is disturbed in Rab7b deficient cells.
There is no mechanistic insight explaining the effects observed by the lack of Rab7b, although correlation between myosin IIA, F-actin and lysosome distribution and organization is described. Authors have not addressed the endogenous localization of Rab7b in their cellular system. Does Rab7b localize to lysosomes, to endosomes, to the trans-Golgi compartment? Is there any change in the localization and structure of these compartments when cells polarize upon LPS stimulus? Specific immune-localization of endogenous protein is required to assess this issue.

Following the reviewers insightful remarks, we have performed additional experiments that now provide a better mechanistic insight. Together with the previous data in the manuscript, the new results indicate that Rab7b recruits myosin II to lysosomes, bringing the motor in close proximity to TRPML1, which activates myosin II at the cell rear, physically linking the late endosomes/lysosomes to the actomyosin cytoskeleton for the regulation of DC motility.
To address the endogenous localization of Rab7b in our cellular system, we have co-stained endogenous Rab7b in DCs together with markers for endosomes/lysosomes and trans-Golgi as suggested. As shown in Supplementary Figure 1, in mature DCs, Rab7b localizes to late endosomes/lysosomes but not to early endosomes nor trans-Golgi. It also colocalizes with myosin II (Supplementary Figure 1) and TRPML1 (Fig 6H). The staining of endogenous Rab7b in immature DCs is not clearly detectable and this is in line with previous studies showing that Rab7b expression increases upon LPS stimulation (Berg-Larsen et al., 2013).

More specific markers for lysosomes should be added for the cell localization studies. Do lysosomes localize to the rear edge of polarized dendritic cells? Are they bound to microtubules or to F-actin? Are microtubules and centrosome correctly located for migration in the Rab7b silenced cells?

We have shown that lysosomes localize to the rear of polarized dendritic cells using WGA (Supplementary Figure 4), which has been demonstrated to co-localize with Lamp1 (Bretou et al., 2017). In the same paper, it was also very nicely demonstrated that lysosomes (stained by both WGA and Lamp-1) localize to the rear of polarized dendritic cells. We have also verified that using Lamp-2 antibody and a figure is included below for the referee.

We have further stained dendritic cells migrating in microchannels for microtubules and F-actin, together with anti-Lamp2 to address whether lysosomes are bound to microtubules or to F-actin. We found that lysosomes are associated to microtubules, as expected, but also with patches of actin, as seen in the figure included below for the referee. The main cluster of lysosomes is localized close to the centrosome/microtubule-organizing center. As suggested by the referee, we

© 2021. Published by The Company of Biologists under the terms of the Creative Commons Attribution License (https://creativecommons.org/licenses/by/4.0/).

verified that the centrosome is correctly located in Rab7b KO DCs and found that, similar to the control cells, more than 95 % of the KO cells had the centrosome positioned at the cell rear (Supplementary Figure 4D- E).

NOTE: We have removed unpublished data that had been provided for the referees in confidence.

Where is myosin light chain phosphorylated in DCs? Localization of myosin light chain (phosphorylated) should be assessed in LPS-matured DC in the presence and the absence of Rab7b. Does this phosphorylation increase with LPS and polarization? Is this affected by Rab7b silencing and therefore may have a role in DC migration?

We have now compared phospho-myosin localization in WT and Rab7b-KO DCs. In line with the role of Rab7b in fast DC migration, we found that LPS-treated Rab7b KO DCs had less phosphorylated myosin at the rear edge, compared to control cells (Figure 6C-D).

Reviewer #2 (Significance (Required)):
This an interesting study, but the findings described are not well connected. It needs additional experimentation to provide a more mechanistic view.

We appreciate the reviewer's comments. We have now performed all the recommended experiments, which strengthen the role of Rab7b as link between lysosomal signaling and actomyosin cytoskeleton regulation in dendritic cells. The new results further provide mechanistic detail by showing that Rab7b is present on late endosomes/lysosomes together with myosin IIA and the lysosomal calcium channel TRPLM1 which is responsible for triggering myosin II phosphorylation.
Most importantly, we now show that Rab7b interacts with TRPML1, and that myosin phosphorylation at the rear edge of migrating DCs is affected by Rab7b. Taken together, this indicates that Rab7b is a link between late endosomes/lysosomes and the actomyosin cytoskeleton by bridging myosin II to TRMPL1 for the local activation of the actin motor at the cell rear.

Reviewer #3 (Evidence, reproducibility and clarity (Required)):
Vestre et al investigate the consequences of the loss of the small GTPase Rab7b on DC migration, using monocyte-derived human dendritic cells (MDDCs) and bone-marrow derived mouse dendritic cells (BMDCs).
They reveal that loss of Rab7b results in (1) a (mild; 20% speed reduction) defect in DC velocity, (2) a defect in DC polarity, (3) an increased macropincocytosis-like vesicular uptake, (4) changes in lysosomal dynamics, (5) reduced nuclear translocation of the lysosome regulator (transcription factor) TFEB, and (6) reduced MLC phosphorylation. The authors convincingly exclude that these effects are a consequence of a defective DC maturation phenotype. The results are interesting, and appear clear and reproducible; especially the DC migration/polarisation phenotype is reproducible by alternative loss-of-function approaches (knockdown and knockout) and alternative migration assays (3D collagen matrices and microchannels). However, the causality between some of the different findings and thus parts of the proposed mechanisms are not entirely clear.

Major Point:
A number of causality arguments in the manuscript build on previous findings from below mentioned manuscripts (see 'significance') and from Vargas at al 2016 (Bretou, M. et al. Lysosome signaling controls the migration of dendritic cells. Science Immunology 2, eaak9573 (2017); Vargas, P. et al.
Innate control of actin nucleation determines two distinct migration behaviours in dendritic cells. Nature cell biology 18, 43-53 (2016).). Yet, the direct causality regarding the different identified Rab7b loss-of-function phenotypes should be clarified by Vestre et al, or at least the causality claims in the manuscript should be changed. The authors write (e.g. abstract line 40) that they identify Rab7b as the missing physical link between lysosomes and the actomyosin cytoskeleton. However the manuscript does not include experiments directly showing this physical link, and also not indirectly (e.g. myosin localisation upon Rab7b depletion; Rab7b localisation). Given the observed defects in nuclear translocation of the transcription factor TFEB in Rab7b depletion DCs, one could speculate that the findings could also be explained by changes in transcriptional regulation (instead of a physical linkage).

© 2021. Published by The Company of Biologists under the terms of the Creative Commons Attribution License (https://creativecommons.org/licenses/by/4.0/).

We thank the reviewer for the thorough and detailed feedback. We have now clarified, performing additional experiments, the direct causality regarding the different identified Rab7b loss-of-function phenotypes. Most importantly, we now demonstrate that Rab7b interacts and colocalizes at the cell rear of migrating DCs, with the lysosomal calcium channel TRPLM1 (Figure 6G-H). Furthermore, we analyzed myosin localization in Rab7b KO DCs and Rab7b localization as suggested.
Immunofluorescence analysis of myosin localization demonstrate that Rab7b KO DCs fail to increase the amount of myosin II at the cell rear after addition of LPS (Figure 4 A-C) and that myosin phosphorylation at the cell rear is reduced in Rab7b KO DCs (Figure 6C-D). We also detected endogenous Rab7b on Lamp-2 positive late endosomes/lysosomes, as well as together with myosin II (Supplementary Figure 1A). Altogether, these results show both directly and indirectly that Rab7b is the missing physical link between lysosomes and the actomyosin cytoskeleton. They further a deeper mechanistic view about the role of Rab7b in the activation of DCs' migration upon microbial sensing: Rab7b recruits myosin II to lysosomes, bringing the motor in close proximity to TRPML1, which activates myosin II at the cell rear, physically linking the late endosomes/lysosomes to the actomyosin cytoskeleton for triggering fast DC motility.

Further, the causality between enhanced macropinocytosis phenotype and reduced cell migration phenotype is not directly shown, as the authors currently only correlate both findings with each other: could the authors perform experiments to directly address the causality that the enhanced macropinocytosis rate in Rab7b KD/KO cells indeed is causative for the reduced migration speed (e.g. does macropinocytosis inhibition in Rab7b KOs rescue the cell migration phenotype)?

The experiment suggested by the reviewer to address the causality between enhanced macropinocytosis rate and reduced migration speed is interesting and we are willing to perform it. However, we cannot rule out that macropinocytosis inhibition would not rescue the cell migration phenotype, as for example, the macropinocytosis inhibitor 5-(*N*-ethyl-*N*-isopropyl)amiloride (EIPA) has been shown to inhibit migration in some circumstances (Lagana et al., 2000; Schneider et al., 2009). It is, however, already known that cell migration and antigen uptake/macropinocytosis are antagonist processes regulated by myosin II localization (Chabaud et al., 2015). In more detail, myosin II enrichment at the front of DCs is required for macropinocytosis and therefore reduces the speed of locomotion. According to the model we propose, Rab7b, by recruiting myosin on lysosomes upon LPS stimulation, depletes the myosin II pool on the front of the cell, reducing the macropinocytic activity and consequently increases cell motility. The new results from the analysis of myosin localization upon Rab7b depletion (Figure 4A-C and 6C-D) confirm this model.

Further Points:
- Line 34: The statement "Rab7b is a key regulator of migration of DCs..." appears exaggerated given the approx. 20% speed reduction of Rab7b KO/KDs.

We have removed the word "key" from this sentence.

- Timing of Rab7b up regulation: the authors state that their previously identified up regulation of Rab7b correlates with an enhanced DC migratory ability (e.g. lines 270-274). Yet, in their previous manuscript the authors showed an early up regulation of Rab7b on RNA and protein level 4 hours after LPS stimulation, a time point where the macropinocytosis activity might rather be high and the migratory activity rather low. At later time points, the RNA and protein levels drop to the original level again, and for the protein level even lower than the unstimulated control. Yet, these are the timepoints where the authors e.g. investigate DC migration/macropinocytosis in the microchannels (up to 20 hours). The authors should clarify this aspect and explain how this aspect fits into their model.

According to the model we suggest, the up regulation of Rab7b in the first hours after LPS stimulation is important for initiating the switch between the high macropinocytic activity characterizing immature DCs and the high migratory activity of mature DCs. Since Rab7b triggers this switch via lysosomal signaling, a high level of Rab7b is required only initially, to promote the switch. At later time points, high levels of Rab7b are not necessary anymore to maintain the established actomyosin reorganization in mature DCs and therefore Rab7b stabilizes to lower

© 2021. Published by The Company of Biologists under the terms of the Creative Commons Attribution License (https://creativecommons.org/licenses/by/4.0/).

levels.
In the absence of Rab7b, the switch between high macropinocytic activity and high migratory activity is inhibited, causing LPS-stimulated cells to retain high macropinocytic activity even 20 hours after LPS stimulation. We agree that the indicated lines may be confusing, and to avoid any misunderstanding, we have therefore removed them.

- Podosomes are adhesive structures rather observed in immature DCs than mature DCs. Yet, the authors identify a reduced migration speed only in mature and not immature DCs. Thus, it is unclear why the authors investigate the polarity of the actin cytoskeleton in mature DCs on highly adhesive PLL-coated coverslips (potentially artificially inducing podosome formation). More appropriate could be the use of less adhesive assays, such as the 'PDMS-confiner' (Maiuri, P. et al. Actin flows mediate a universal coupling between cell speed and cell persistence. Cell 161, 374-386 (2015).) or the under- agarose assay that is more simple to establish in every laboratory (Heit, B. & Kubes, P. Measuring Chemotaxis and Chemokinesis: The Under-Agarose Cell Migration Assay. Sci. Signal. 2003, pl5-pl5 (2003).).

The change in podosome distribution in mature DCs was the very initial observation that motivated us to further continue this research. If the reviewer thinks that this information is not necessary, we will remove it. Indeed, we agree that this is not the most appropriate system to study the polarity of the actin cytoskeleton and this is why we later investigated actin distribution and cell migration using micro-fabricated channels. These channels are a system widely used to study DC polarization and motility as, similarly to 'PDMS-confiner', are a less adhesive system that confines the cells.
Importantly, the experiments using microchannels confirmed the defects in polarization in absence of Rab7b (Figure 4).

- Line 151: It is written 'Rab7b KO DCs', however, the corresponding figure indicates that Rab7b-depleted (and not knockout) cells have been used in this experiments. These authors should check whether the experimental details on knockout versus knockdown have been accurately described in all figures, legends, and text paragraphs.

We apologize for this mistake and thank the referee for pointing it out. We have corrected this, and gone through all other parts of the manuscript to check that there are no other similar mistakes.

- Line 174 and corresponding Figure: it is stated that the fraction of actin located at the cell front is 30% higher in Rab7b KO DCs. However, the data in Fig. 4B rather show an equal actin signal in front; the ratio seems to be only changed due to a decreased actin signal in the cell back. The authors should clarify this aspect and explain how this finding fits into their model.

In Fig. 4B (Fig. 4D in the revised version of the manuscript) we have generated density maps that show the average distribution of actin. The maps were generated by projecting the mean signal of all individual cells. Thus, the map is not showing one individual cell, but an average of several cells. The colors of the map represent the relative intensity levels in each map. However, as the minimum and maximum values for the lookup tables (LUT) are different for WT and KO, the colors in each map do not represent the same intensity values for WT and KO. This means that the red in the WT map does not correspond to the same intensity value as the red in the KO map. As pointed out by the referee this may give the impression that the actin signal is equal in the front.
To make this clearer, we provide below a figure where both maps have the same minimum and maximum values for the LUT, so that the color corresponds to the same intensity value in both maps.

NOTE: We have removed unpublished data that had been provided for the referees in confidence.

What we aim to show in this experiment is that the relative difference of actin distribution between the front and back is higher for KO cells. This suggests that in the KO cells a major fraction of the total cellular actin is retained at the front for macropinocytosis, which is supported by the results presented in Fig. 5A-D, showing that the macropinocytic activity in the Rab7b KO DCs is higher compared to WT DCs, thus contributing to retain a bigger fraction of the total cellular

© 2021. Published by The Company of Biologists under the terms of the Creative Commons Attribution License (https://creativecommons.org/licenses/by/4.0/).

actin at the front. In WT cells, instead, actin is also at the rear as its polymerization is necessary to generate pressure against the channel walls for migration. Even though the macropinocytic activity in the WT DCs decreases, some cells are still doing some macropinocytosis, and this also contributes to the actin signal at the front in the average image.

- The authors use the distribution of actin and the vesicle volume as a read out for the efficiency of macropinocytosis. Macropinocytosis research typically takes advantage of large labeled dextran (70kDa) to specifically investigate macropinocytosis (e.g. Commisso C, Flinn R J and Bar-Sagi D 2014 Determining the macropinocytic index of cells through a quantitative image-based assay. Nature Protocols 9 182-92). Thus, it is surprising that the authors used a 10kDa dextran that could also be taken up by other endocytotic pathways. Moreover, the authors could control whether the indeed observe macropinocytosis by using a macropinocytosis inhibitor like EIPA. Further, it is surprising to see that the intra-vesicular dextran intensity appears equal in the depicted images, yet, a characteristic of macropinocytosis-vesicles is their maturation that goes in hand with volume shrinkage and thus fluid-concentration (increase in dextran intensity; e.g. Freeman S A, Uderhardt S, Saric A, Collins R F, Buckley C M, Mylvaganam S, Boroumand P, Plumb J, Germain R N, Ren D and Grinstein S 2020 Lipid-gated monovalent ion fluxes regulate endocytic traffic and support immune surveillance Science 367 301-5)

During initial experiments, we tested 70kDa dextran. However, due to problems with the formation of aggregates/crystals we did not find this marker suitable for our experiments in microchannels. We thus used 10kDa dextran, as it has previously been used in experiments on dendritic cells in microchannels (Chabaud et al., 2015; Moreau et al., 2019). In the study by Chabaud et al., they also demonstrated that EIPA inhibition of macropinocytosis impaired the uptake of 10kDa dextran at the cell front.
Regarding the dextran intensity, we intentionally increased the intensity to make all the macropinosomes clearly visible, so the fine intensity differences are difficult to identify in our images. As the aim of our experiment was not to study intensity changes upon macropinosome maturation, our settings were not adjusted for this type of study. Most importantly, in Freeman et al., a dextran pulse was followed by wash for an accurate tracking of the intensity of maturing macropinosomes, while in our experiments we were interested in the bulk macropinocytosis, and therefore the dextran was retained in the channels to ensure a continuous uptake. Nevertheless, we do observe the events mentioned by the reviewer in some cases, as illustrated in the figure provided below where it is highlighted a macropinosome that shrinks and at the same time the mean intensity of its dextran content increases.

NOTE: We have removed unpublished data that had been provided for the referees in confidence.

- Figure 4D and E: The quantification in E shows an approx. doubling in vesicle area in Rab7b KOs, yet the image in D shows a much extremer difference. It would be recommended to use a representative image for Figure 4D.

We agree with the reviewer and replaced the image with a more representative one (Fig. 5A in the revised manuscript).

- Figure 4G: According to the methods section, this analysis has been performed with images in 1min intervals. Thus, it appears rather inappropriate to show a scale in seconds; given the small in difference of roughly 20sec, it is unclear whether this experimental setup can really infer the conclusion (longer vesicle lifetime).

The first two experiments were performed with 30 second intervals, and gave a difference of 26 % (experiment 1) and 28 % (experiment 2). For the third experiment, due to technical problems, we were only able to image with 1 minute intervals. However, the difference was still similar to the first two experiments (30 %). Our main aim with this analysis is to illustrate the difference between the macropinosomes in WT and KO cells as we observed that the macropinosomes typically disappeared shortly after formation in the WT cells, while they persisted longer in the KO cells. Although we agree that shorter time intervals would have given a more accurate determination of macropinosome lifetime, this would have limited the amount of cells we were able to image, as they were imaged using multi-field positioning mode in order to track multiple cells simultaneously

© 2021. Published by The Company of Biologists under the terms of the Creative Commons Attribution License (https://creativecommons.org/licenses/by/4.0/).

with the same incubation time for dextran.
To generate less confusion, we have now presented the data as relative to control instead of presenting them in seconds (Fig. 5D in the revised manuscript).

- Figure 5A/B: the graph in B only quantifies the number of small lysosomes, finding a reduction in Rab7b KO DCs. However, the picture in A indicates that there is actually an increase in overall lysosome staining in Rab7b KO DCs. It is unclear why the authors do not quantify and comment on this aspect.

We analyzed the total area of lysosomes, but did not detect a significant difference. The data is now included in Supplementary Figure 4A. We have therefore replaced the image in figure 5A (now Fig. 5E) with a more representative one for Rab7b KO.

- Statistics: the authors frequently show s.e.m.; thereby, however, error bars get smaller with higher n numbers. To exclude this effect in datasets with high n numbers, it is recommended to depict e.g. the 95% confidence interval (CI; e.g. Cumming, G., Fidler, F. & Vaux, D. L. Error bars in experimental biology. J Cell Biology 177, 7-11 (2007).). Moreover, it is unclear why some graphs do not show any SD/SEM/CI (E.g. Fig 1C, 3C, 3D, 3F). Further, it would be helpful to plot individual datapoints into the bar graphs to be able to judge the spread of the data.

We have changed the graphs trying to take into account both this reviewer's suggestions and reviewer 1 suggestions. In order to satisfy both reviewers we replaced s.e.m. bars with s.d. and plotted individual datapoints where appropriate.

Reviewer #3 (Significance (Required)):
Dendritic cells (DCs) link the innate and adaptive immune system by their ability to migrate from peripheral tissue locations (e.g. skin) to secondary lymphoid organs (lymph nodes). Whereas several regulators of DC migration have been already identified (e.g. actin cytoskeletal regulators) the field clearly lacks an understanding of additional molecular pathways responsible for DCs migration. In the accompanying manuscript, Vestre et al investigate how the loss of the small GTPase Rab7b affects DC migration in vitro.

In previous papers, the authors already showed that
1) Rab7b interacts with myosin II in human DCs (using immunopreciptation and co-localisation) and alters actin stress fibre formation, myosin light chain (MLC) phosphorylation, cell spreading and cell migration in HeLa cells (Borg, M., Bakke, O. & Progida, C. A novel interaction between Rab7b and actomyosin reveals a dual role in intracellular transport and cell migration. J Cell Sci 127, 4927-4939 (2014).)
2) Rab7b is up up-regulated on RNA and protein level in DCs upon LPS simulation (Berg-Larsen, A., Landsverk, O. J. B., Progida, C., Gregers, T. F. & Bakke, O. Differential Regulation of Rab GTPase Expression in Monocyte-Derived Dendritic Cells upon Lipopolysaccharide Activation: A Correlation to Maturation-Dependent Functional Properties. Plos One 8, e73538 (2013).).
Building on these results, Vestre et al investigate now the consequences of the loss of the small GTPase Rab7b on DC migration.

Building on the papers mentioned by the reviewer, our present study provides novel evidence addressing the mechanistic link between lysosomes and the actomyosin cytoskeleton in DCs, which is still poorly defined.
We now reveal that Rab7b, by interacting also with the lysosomal calcium channel TRPML1, regulates actomyosin recruitment and activation at the rear of migrating mature DCs, therefore functioning as the mechanistic link between lysosomal signaling and activation of the actomyosin cytoskeleton to ensure directed migration.

Berg-Larsen, A., O.J.B. Landsverk, C. Progida, T.F. Gregers, and O. Bakke. 2013. Differential Regulation of Rab GTPase Expression in Monocyte-Derived Dendritic Cells upon Lipopolysaccharide Activation: A Correlation to Maturation-Dependent Functional Properties. *PLoS ONE*. 8:e73538.
Chabaud, M., M.L. Heuze, M. Bretou, P. Vargas, P. Maiuri, P. Solanes, M. Maurin, E. Terriac, M. Le Berre, D. Lankar, T. Piolot, R.S. Adelstein, Y. Zhang, M. Sixt, J. Jacobelli, O. Benichou, R.

© 2021. Published by The Company of Biologists under the terms of the Creative Commons Attribution License (https://creativecommons.org/licenses/by/4.0/).

Voituriez,
M. Piel, and A.M. Lennon-Dumenil. 2015. Cell migration and antigen capture are antagonistic processes coupled by myosin II in dendritic cells. *Nat Commun*. 6:7526.
Lagana, A., J. Vadnais, P.U. Le, T.N. Nguyen, R. Laprade, I.R. Nabi, and J. Noel. 2000. Regulation of the formation of tumor cell pseudopodia by the Na+/H+ exchanger NHE1. *Journal of Cell Science*.
113:3649-3662.
Moreau, H.D., C. Blanch-Mercader, R. Attia, M. Maurin, Z. Alraies, D. Sanséau, O. Malbec, M.-G. Delgado, P. Bousso, J.-F. Joanny, R. Voituriez, M. Piel, and A.-M. Lennon-Duménil. 2019.
Macropinocytosis Overcomes Directional Bias in Dendritic Cells Due to Hydraulic Resistance and Facilitates Space Exploration. *Developmental Cell*. 49:171-188.e175.
Schneider, L., C.-M. Stock, P. Dieterich, B.H. Jensen, L.B. Pedersen, P. Satir, A. Schwab, S.T. Christensen, and S.F. Pedersen. 2009. The Na+/H+ exchanger NHE1 is required for directional migration stimulated via PDGFR-alpha in the primary cilium. *The Journal of cell biology*. 185:163- 176.

Original submission

First decision letter

MS ID#: JOCES/2021/259221

MS TITLE: Rab7b regulates dendritic cell migration by linking lysosomes to the actomyosin cytoskeleton

AUTHORS: Katharina Vestre, Irene Persiconi, Marita Borg Distefano, Nadia Mensali, Noemi Antonella Guadagno, Marine Bretou, Sebastien Walchli, Catharina Arnold-Schrauf, Oddmund Bakke, Marc Dalod, Ana-Maria Lennon-Dumenil, and Cinzia Progida
ARTICLE TYPE: Research Article

Thank you for sending your manuscript to Journal of Cell Science through Review Commons.

I am happy to tell you that your manuscript has been accepted for publication in Journal of Cell Science, pending standard ethics checks.

Reviewer 1

Advance summary and potential significance to field

The authors have further improved their initial manuscript with additional experiments and discussion that bring together all the evidence presented herein.
The link between actomyosin, lysosomes through the Rab7b-TRPML1 connection is nicely shown now.

Comments for the author

Authors have therefore fulfilled the comments and suggestion raised during the first round of revision. I recommend acceptance of the manuscript as it is.
As a minor point, some editing should be performed (e.g.: “next next” at line 186).

Reviewer 2

Advance summary and potential significance to field

© 2021. Published by The Company of Biologists under the terms of the Creative Commons Attribution License (https://creativecommons.org/licenses/by/4.0/).

The authors investigate the role of the small GTPase Rab7b during dendritic cell migration. The work builds up on previous data, which demonstrate that Rab7b interacts with myosin II in human dendritic cells and modulates actin stress fiber formation, myosin phosphorylation and cell migration in HeLa cells (Borg et al., JCS, 2014). In the present study they address the missing link between lysosomes and the actomyosin cytoskeleton in dendritic cells and provide evidence that Rab7b mediates actomyosin recruitment and activation at the uropod of migrating dendritic cells thus ensuring directional locomotion.
Their findings are of great interest for the field of cell biology as well as immunology as they contribute to a better understanding of the molecular pathways mediating cell migration, which are still ill defined.

Comments for the author

The authors have made substantial improvements to the manuscript and addressed all my concerns. I strongly recommend publication of the manuscript in JCS.

© 2021. Published by The Company of Biologists under the terms of the Creative Commons Attribution License (https://creativecommons.org/licenses/by/4.0/).
